# Features and evolutionary adaptations of the mitochondrial genome of *Garuga forrestii* W. W. Sm

**DOI:** 10.3389/fpls.2024.1509669

**Published:** 2025-01-20

**Authors:** Rong Chen, Rui Rao, Chun Wang, Dongbo Zhu, Fen Yuan, Liangliang Yue

**Affiliations:** ^1^ Yunnan Key Laboratory of Plateau Wetland Conservation, Restoration and Ecological Services, Southwest Forestry University, Kunming, China; ^2^ National Plateau Wetlands Research Center, Southwest Forestry University, Kunming, China; ^3^ National Wetland Ecosystem Fixed Research Station of Yunnan Dianchi, Southwest Forestry University, Kunming, China; ^4^ Dianchi Lake Ecosystem Observation and Research Station of Yunnan Province, Kunming, China

**Keywords:** *Garuga forrestii*, mitochondrial genome, phylogenomic, adaptation, repetitive sequence

## Abstract

**Introduction:**

*Garuga forrestii* W. W. Sm. is a tree species of the Burseraceae family, endemic to China, found in hot/warm-dry valleys. This species plays a crucial role in maintaining biodiversity in these ecosystems.

**Methods:**

We performed *de novo* assembly of the *Garuga forrestii* mitochondrial genome using PMAT (v.1.5.4), resulting in a typical circular molecule of 606,853 bp. The genome consists of 31 tRNA genes, 3 rRNA genes, 35 protein-coding genes, and 1 pseudogene. The study also investigates RNA editing sites and evolutionary patterns.

**Results:**

The mitochondrial genome exhibits a low proportion of repetitive sequences (3.30%), suggesting a highly conserved structure. A high copy number of the *trnM-CAT* gene (4 copies) is noted, which may contribute to genomic rearrangement and adaptive evolution. Among the 476 RNA editing sites, hydrophilic-hydrophobic and hydrophobic-hydrophobic editing events are most common, accounting for 77.10%. Negative selection predominates among most genes (Ka/Ks < 1), while a few genes (e.g., *matR*, *nad3*, *rps1*, *rps12*, and *rps4*) show signs of positive selection (Ka/Ks > 1), potentially conferring evolutionary advantages. Additionally, a significant A/T bias is observed at the third codon position. Phylogenomic analysis supports the APG IV classification, with no evidence of horizontal gene transfer.

**Discussion:**

This mitochondrial genome offers valuable insights into the adaptive mechanisms and evolutionary processes of *Garuga forrestii*. It enhances our understanding of the species' biogeography in tropical Southeast Asia and Southwest China, providing key information on the evolutionary history of this genus.

## 1 Introduction

*Garuga forrestii*, Burseraceae, is a deciduous tree species up to more than 25 meters high, endemic to southwest China, thrives in valley habitats at elevations ranging from 900 to 2400 meters above sea level, sometimes dominates the upper slopes of savannas in river valleys (Tiamiyu et al., 2021). This species is primarily distributed in the warm valleys of Sichuan and Yunnan provinces, particularly along the Jinshajiang, Yangbijiang, Lancangjiang, Honghe rivers, and their major tributaries. Remarkably, *G. forrestii* represents the sole species of the Burseraceae family found beyond the Tropic of Cancer, with its northernmost distribution reaching latitudes above 28°N in Leibo, Sichuan. The species flowers in the period between March and April, preceding the onset of the monsoon season, with fruiting occurring from May to September. *G. forrestii* has been identified as a promising candidate for ecological restoration, particularly for riparian vegetation rehabilitation and riverbank stabilization in valley environments. The fruits of the tree serve as an important food source for a variety of avian and mammalian species, thus playing a role in maintaining faunal population sizes. Despite its ecological importance, a recent phylogenomic study of *G. forrestii* and other related species within the Burseraceae family revealed intricate evolutionary patterns, with notable discrepancies between plastid and nuclear genomic datasets (Zhu et al., 2024). However, mitochondrial genomic data for this species remain absent, representing a significant gap in current research and a promising direction for future investigation.

Mitochondria play crucial roles in plant development and reproduction by participating in energy production, metabolism, and maintaining cellular homeostasis through their semi-autonomous genetic system (He et al., 2020; Ke et al., 2023). Each plant species has a unique mitochondrial genome, known for extreme variations in size, structural complexity, mutation rates, as well as its ability to absorb foreign DNA (Petersen et al., 2020). These differences are primarily attributable to repetitive sequences, including elements resulting from horizontal gene transfer (HGT), introns, and DNA segments transferred from chloroplast (cp) and nuclear genomes (Gandini et al., 2019; Tanaka et al., 2012).

Horizontal gene transfer (HGT), particularly involving transfers between mitochondria, is now recognized as a significant driver of evolution in land plant (Yue et al., 2012; Gandini and Sanchez-Puerta, 2017). Pant mitochondrial genomes exhibit structural complexity and size variation, while their protein-coding genes evolve slowly and are less susceptible to substitution saturation, making them ideal for phylogenetic studies in higher taxonomic categories (Dong et al., 2022). However, most of the mitochondrial genomes are characterized by abundant repeat sequences which facilitate recombination and contribute to dynamic structural rearrangements, such as the formation of subgenomic circles and isomers (Guo et al., 2024; Feng et al., 2024). An example of this complexity is observed in *Gastrodia elata*, whose mitochondrial genome contains 19 subgenomes, including 12 circular and 7 linear forms (Yuan et al., 2018).

Frequent recombination and the high prevalence of simple sequence repeats (SSRs) drive the structural diversity observed in plant mitochondrial genomes. SSRs also serve as markers for studying genetic diversity and species identification (Ma et al., 2016), and homologous recombination involving these repeats has been documented across numerous plant species (Mower et al., 2012; Xu et al., 2013). Recombination between primary and secondary conformations further complicates sequencing, particularly in angiosperms.

Another important feature of mitochondrial genomes is RNA editing, which refers to the programmed modification of the RNA nucleotide sequence relative to the corresponding DNA sequence. This process encompasses two main types of nucleotide changes: insertion/deletion and substitution (Goremykin et al., 2008). This process is widespread and highly variable in mitochondria (Bi et al., 2016). These RNA modifications, which often result in non-synonymous substitutions at the first and second codon positions, can influence gene expression and impact phylogenetic analyses (Dong et al., 2022; Goremykin et al., 2008).

In this study, we presented the first complete mitochondrial genome of *G. forrestii*, provide a comprehensive analysis of its features, including the proportions of repetitive sequences, RNA editing, codon usage, collinearity, gene transfers from chloroplasts to mitochondria, nucleotide diversity, selection pressures, and phylogenomic relationships. The primary objectives of this study were: 1) to characterize the mitochondrial genome of *G. forrestii*, and 2) to elucidate the evolutionary dynamics of its mitochondrial genome within the context of the Sapindales order.

## 2 Materials and methods

### 2.1 Plant material and DNA extraction

Fresh leaves of *G. forrestii* were collected from Yangwu, Xinping County, Yunnan, China (102°7′41.75″E, 24°1′54.84′N). A voucher specimen (yue187 Specimen No. 202382702-2) was deposited at the College of Wetland, Southwest Forestry University, Kunming, China (email: mcm18213835817@126.com). The mitochondrial genome data of *G. forrestii* was generated by Biomarker Technologies (www.biomarker.com.cn) using the PacBio HiFi (CCS) library construction and sequencing service. Through PacBio third-generation sequencing and post-sequencing quality control, a total of 15,498,150,008 bp of CCS data was obtained. Transcriptome sequencing yielded 34.60 million reads, with a total of 10.37 Gb of clean data. The average data output per sample was 10.37 Gb, with a Q30 base percentage of 93.37% or higher.

### 2.2 Mitochondrial genome assembly and annotation

We performed *de novo* assembly of the *G. forrestii* mitochondrial genome using PMAT (v.1.5.4) (Bi et al., 2024) with the command PMAT autoMito -I -o -st -g -m. Genome visualization was carried out using Bandage (v0.8.1) (Wick et al., 2015). To ensure a pure mitochondrial genome assembly, chloroplast and nuclear genome fragments were manually removed using the command PMAT graphBuild -c -a -gs -rs -o -s, resulting in a graphical representation of the mitochondrial genome.Annotation of the *G. forrestii* mitochondrial genome was conducted using coding gene sequences from Arabidopsis thaliana (NC037304.1) as references. Protein-coding genes (PCGs) were identified using the Geseq tool (Tillich et al., 2017; https://chlorobox.mpimp-golm.mpg.de/geseq.html) and IPMGA (http://www.1kmpg.cn/ipmga/). For tRNA annotation, we used tRNAscan-SE (v.1.4) (Lowe and Eddy, 1997) with default parameters, while rRNA annotation was performed using BLASTN (v.2.10.1) (Chen et al., 2015). Manual corrections to the annotation were made using Apollo (v.1.11.8) (Lee et al., 2009) to address any errors detected during the process (Darling et al., 2019).The mitochondrial genome map was generated using OGDRAW (v.1.3.1) (https://chlorobox.mpimp-golm.mpg.de/ogdraw.html). Subsequently, the chloroplast genome of *G. forrestii* was assembled using Oatk (v.1.0) (https://github.com/c-zhou/oatk) and annotated with Geseq. Both the annotated mitochondrial and chloroplast genomes of *G. forrestii* have been deposited in GenBank.

### 2.3 Detection of RNA editing events in *G. forrestii* mitogenome

we extracted the coding sequences (CDs) of each protein-coding gene (PCG) with 100 bp-long flanking regions as reference sequences (Yang et al., 2023). We then mapped strand-specific RNA-seq reads to these reference sequences using HISAT2 (v 2.2.1) (Chen et al., 2015; Kim et al., 2015), with the parameters “–rna-strandness RF –sensitive –no-mixed –no-discordant” (The minicircular and extremely heteroplasmic mitogenome of the holoparasitic plant *Rhopalocnemis phalloides*). DNA sequencing reads were mapped to the reference sequences extracted above using BWA (v0.7.12-r1039) (Li and Durbin, 2009), and REDItools v2 was used to detect RNA editing sites based on the mapping results (Picardi and Pesole, 2013). Finally, REDItools (v 2.0) was used to extract unidentified SNP sites, and IGV software (v 2.15.1) (Milne et al., 2010) was used to visualize the positioning results of RNA editing sites.

### 2.4 Analysis of relative synonymous codon usage and repeated sequences

We used PhyloSuite (v1.2.2) (Zhang et al., 2019) to extract protein-coding sequences (PCGs) from the mitochondrial genome of G. forrestii. Codon usage bias in the PCGs of *G. forrestii* and H. rhamnoides was analyzed, with relative synonymous codon usage (RSCU) values calculated using MEGA (v7.0.26) (Kumar et al., 2016). These values were then visualized for intuitive interpretation using the RNArtist web platform (https://github.com/fjossinet/RNArtist).The repeat sequences in the mitochondrial genome of *G. forrestii* were identified using the MISA online tool. SSR frequencies were determined based on the criteria set by Beier et al. (2017), with the minimum repeat numbers defined as 10, 5, 4, 3, 3, and 3 for mono-, di-, tri-, tetra-, penta-, and hexanucleotide repeats, respectively. Tandem repeats exceeding six base pairs were detected using Tandem Repeats Finder (v4.09) (Benson, 1999) with a match score threshold of 95%. Dispersed repeats were identified using BLASTN (v2.10.1) (Chen et al., 2015), applying a word size of 7 and an E-value threshold of 1e-5. The spatial distribution of all repeat types was visualized using Circos (v0.69-5).

### 2.5 Homologous fragment analysis and nucleotide diversity analysis

Homologous gene sequences from mitochondrial genomes of various species were aligned using MAFFT (v7.310) (Katoh and Standley, 2013) with default parameters. Fragments exhibiting significant variation were identified, and highly divergent sequences from each mitochondrial genome were extracted using Geneious (v9.0.2) (Kearse et al., 2012). To identify the most similar sequences for ancestral tracing, nucleotide BLAST searches were conducted on these sequences using the online BLAST tool (https://blast.ncbi.nlm.nih.gov/Blast.cgi), optimized for highly similar matches. Ultra-divergent loci with poor alignment quality were filtered using Gblocks (Castresana, 2000) in accordance with the PhyloSuite pipeline (v1.2.2) (Zhang et al., 2019). Gblocks filtering parameters were set to require at least 10 sequences per block and a maximum of 8 consecutive non-conserved positions. Homologous fragments in the mitochondrial and chloroplast genomes of *G. forrestii* were analyzed using BLASTN (v2.7.1+) (Chen et al., 2015), with homologous sequences identified based on a minimum similarity of ≥70% and an E-value threshold of ≤1e-5. The filter length thresholds were set at ≥30 bp for comparisons between chloroplasts and mitochondria, and ≥1000 bp for comparisons involving mitochondria and the whole genome, following the criteria described by Cheng et al. (2023).The identified homologous sequences were visualized using Circos (v0.69-5) (http://circos.ca/software/download/). Nucleotide diversity (Pi) was calculated using DnaSP (v5) (Librado and Rozas, 2009) with sliding window analysis parameters of 100–200 bp, overlapping windows of 10–20 bp, and nucleotide diversity as the chosen statistical measure.

### 2.6 Comparative genomic analysis

In this study, we performed collinearity analysis on *G. forrestii* to examine its mitochondrial genome, while acknowledging the limited available data for the Burseraceae family, and even for Sapindales as a whole. To enhance the reliability of our analysis, we selected eight fully annotated mitochondrial genomes from closely related species within the Sapindales order. Focusing on these closely related species allowed for a more accurate identification of conserved genomic structures and evolutionary patterns. In contrast, the inclusion of distantly related species would have introduced greater variability, complicating the interpretation of collinearity features. The species included in this analysis are *Mangifera indica* (NC035239.1), *Anacardium occidentale* (NC035235.1), *Nitraria tangutorum* (MK431824.1), *Peganum harmala* (MK431826.1), *Sapindus mukorossi* (NC050850.1), *Handeliodendron bodinieri* (NC054241.1), *Citrus unshiu* (NC057142.1), and *Citrus sinensis* (NC037463.1). These species were chosen to represent key evolutionary lineages within the Sapindales, providing a comprehensive view of mitochondrial genome structure and evolution across the order.

To investigate the molecular evolution of the mitochondrial genomes, we calculated the Ka (nonsynonymous substitutions), Ks (synonymous substitutions), and the Ka/Ks ratio for each gene. The Ka/Ks ratio is a critical metric for understanding evolutionary dynamics at the molecular level (Yang and Nielsen, 2000; Zhang et al., 2006). A Ka/Ks ratio of 1 indicates neutral selection, where mutations do not affect the fitness of the organism. A Ka/Ks ratio greater than 1 (Ka/Ks > 1) suggests positive selection, indicating that the gene has undergone adaptive evolutionary changes. Conversely, a Ka/Ks ratio less than 1 suggests purifying selection (Hurst, 2002; Wang et al., 2010), where deleterious mutations are removed from the gene pool by natural selection. The calculated Ka/Ks values were then visualized using box plots generated with the R package ggplot2 (Wickham, 2011), providing a clear representation of the selection pressures acting on each gene. To analyze gene sequence similarities and differences across various species, MUMmer (version 4.0.0beta2) (Darling et al., 2018) was used to generate dot plots for sequence alignment, aligning the *G. forrestii* genome with those of other species using the maxmatch parameter. BLASTN (version 2.10.1) was employed to identify homologous sequences between *G. forrestii* and other species, with parameters set to a word length of 7 bases, an E-value threshold of 1e-5, and a minimum alignment length of 300 bp to illustrate homologous relationships in the figures.

### 2.7 Phylogenetic analysis

The mitochondrial phylogenomic tree was reconstructed using 15 samples, including *G. forrestii* and other 12 species from Sapindales (**Supplementary Table 1**) and *Prunus schneideriana* (NC065066.1) and *Eriobotrya japonica* (NC045228.1) from the Rosaceae were selected as outgroups. The sequences of these mitochondria were aligned using MAFFT (v. 7.310) (Katoh and Standley, 2013). A maximum likelihood (Atkins et al., 2020) tree was reconstructed using the software IQ-TREE 2 (Lanfear et al., 2020) following a substitution model selected automatically by IQ-TREE 2 with an ultra-bootstrap simulation of 1000 replications. The phylogenomic tree was visualized using Figtree (v. 1.4.4) (http://tree.bio.ed.ac.uk/software/figtree/) (Poon et al., 2010).

## 3 Results

### 3.1 Features of the *G. forrestii* mitochondrial genome

**Figure 1:**
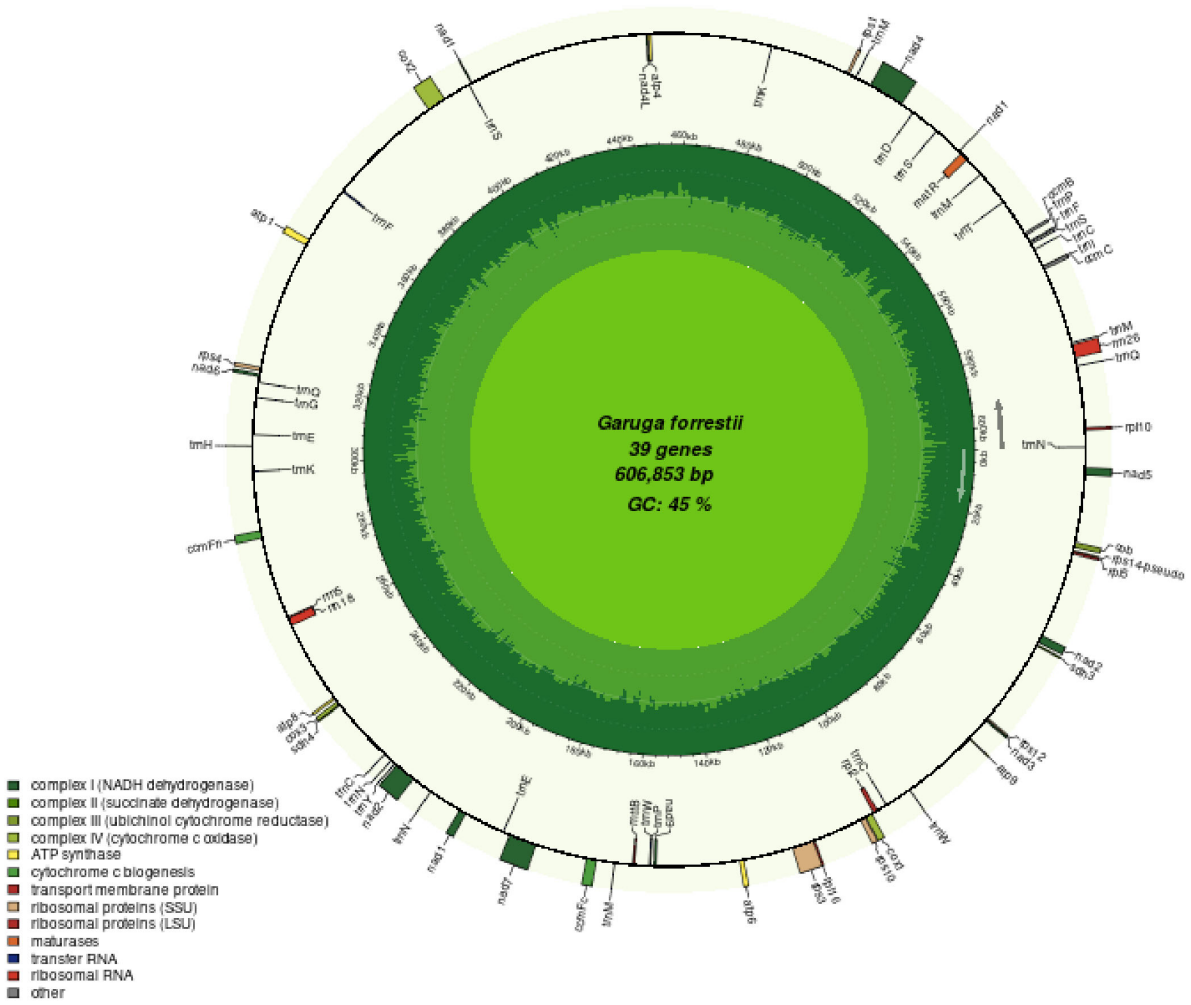
Mitochondrial genome map of *G. forrestii*. Genes transcribed in the clockwise direction are located on the inner circle, while those transcribed counterclockwise are on the outer circle. The innermost light green ring indicates the GC content across the genome.

We generated 13G data from the PacBio platform to assemble the mitochondrial genome of *G. forrestii*. The mitochondrial genome of *G. forrestii* is a circular molecule of 606,853 bp in length (**Figure 1**). The genome has a GC content of 44.77% and an AT content of 55.23%. A total of 70 genes were identified, including 31 tRNA genes (2,307bp), 3 rRNA genes (5,174bp), 35 protein-coding genes (PCGs) (31,065bp), and 1 pseudogene (7,481bp). The mitochondrial genome was deposited onto GenBank with the accession number of CRR1274107.

The encoded proteins can be divided into 26 core mitochondrial genes (which typically refer to genes that are directly involved in mitochondrial energy production and basic biological functions) and 9 non-core mitochondrial genes. The core mitochondrial genome of *G. forrestii* includes 5 ATP synthases, 4 cytochrome c biosynthesis enzymes, 1 ubiquinol cytochrome c reductase, 3 cytochrome c oxidases, 9 NADH dehydrogenases, 1 maturation enzyme, 1 membrane transport protein, and 2 succinate dehydrogenases. The non-core mitochondrial genes consist of 4 large subunit ribosomal proteins (LSU) and 5 small subunit ribosomal proteins (SSU). The mitochondrial genome of *G. forrestii* exhibited multiple copies of several genes (**Table 1**). There were 4 copies of *trnM-CAT* in Transfer RNAs, 3 copies each of *trnC-GCA* and *trnN-GTT*, and 2 copies each of *trnE-TTC*, *trnK-TTT*, *trnP-TGG*, and *trnQ-TTG*. we identified multiple introns across all mitochondrial genes, including 4 introns in NADH dehydrogenase (*nad1*, *nad2, nad5, nad7*), 3 introns in NADH dehydrogenase (*nad4*), and 1 intron each in Cytochrome c biogenesis (*ccmFc*), Cytochrome c oxidase (*cox2*), Ribosomal proteins (*rps10, rps3*), and Transfer RNAs (*trnF-GAA, trnI-TAT*). a pseudogene of Small Subunit Ribosomal Protein (*rps14*) was also observed.

**Table 1:** Gene composition in the mitogenome of *G. forrestii.*.

Group of genes	Gene name
ATP synthase	*atp1 atp4 atp6 atp8 atp9*
Cytohrome c biogenesis	ccmB ccmC ccmFc* ccmFn
Ubichinol cytochrome c reductase	*cob*
Cytochrome c oxidase	cox1 cox2* cox3
Maturases	*matR*
Transport membrance protein	*mttB*
NADH dehydrogenase	nad1**** nad2**** nad3 nad4*** nad4L nad5**** nad6 nad7**** nad9
Large Subunit Ribosomal Proteins (LSU)	*rpl10 rpl16 rpl2 rpl5*
Small Subunit Ribosomal Proteins (SSU)	#rps14 rps1 rps10* rps12 rps3* rps4
Succinate dehydrogenase	*sdh3 sdh4*
Ribosomal RNAs	*rrn18 rrn26 rrn5*
Transfer RNAs	trnC-GCA (3) trnD-GTC trnE-TTC (2) trnF-GAA trnF-GAA* trnG-GCC trnH-GTG trnI-TAT* trnK-TTT (2) trnM-CAT (4) trnN-GTT (3) trnP-TGG (2) trnQ-TTG (2) trnS-GCT trnS-GGA trnS-TGA trnT-GGT trnW-CCA (2) trnY-GTA

* represents one intron, *** represents three introns, and **** represents four introns. #Gene: Pseudo gene; Gene (2): Number of copies of multi-copy genes. 1 represents one intron, **2 represents two introns, ***3 represents three introns, and ****4 represents four introns, and so on.

### 3.2 Repetitive sequence and codon usage analysis

**Figure 2:**
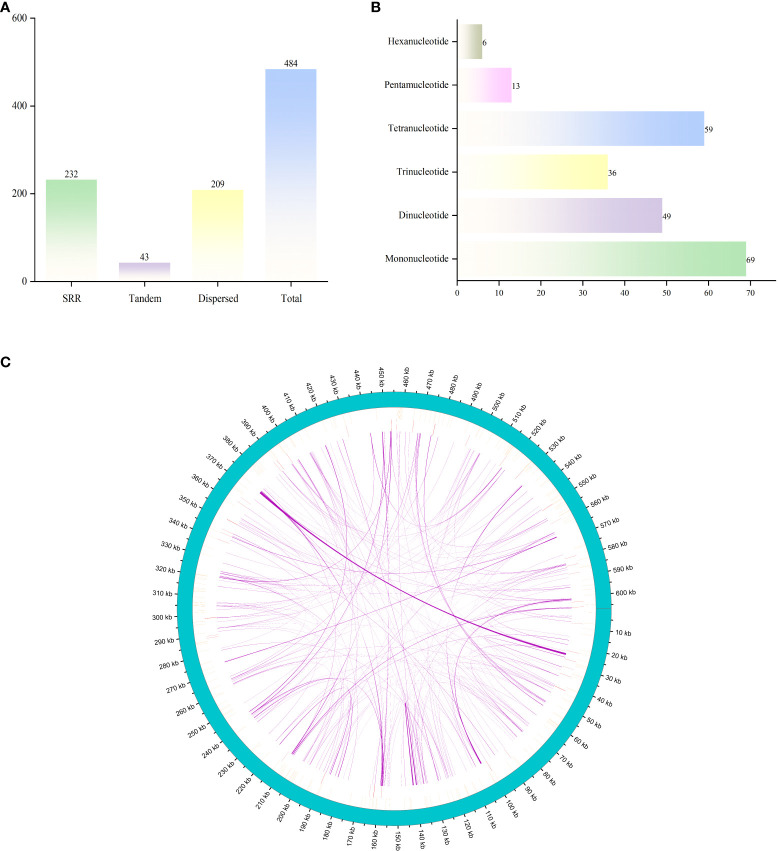
Distribution of repetitive sequences in the mitochondrial genome of *G. forrestii.* **(A)** Types and numbers of repetitive sequences. **(B)** Distribution of simple sequence repeats. **(C)** Positions of repetitive sequences along the genome. The outermost sky-blue circle represents the genome scale. Simple repeats are shown in blue, tandem repeats in red, and dispersed repeats in the innermost circle.

A total of 484 SSR loci were identified (**Figure 2A**), including 232 simple repeats, 209 dispersed repeat sequences, and 43 tandem repeat sequences. The total length of the repeat sequences is 20,041 bp, account for 3.30% of the mitochondrial genome. The total length of tandem repeats is 910 bp, comprising only 0.15% of the mitochondrial genome. Among the different types of repeat sequences, tandem repeats are the least common at 8.88%, while simple repeats are the most prevalent, comprising 47.93%. We identified 232 SSR loci in the *G. forrestii* mitochondrial genome: 69 monomers, 49 dimers, 36 trimers, 59 tetramers, 13 pentamers, and 26 hexamers (**Figure 2B**). Monomers and tetramers were the most abundant, together accounting for 55.17% of the total SSRs. Pentamers and hexamers were the least frequent, comprising only 8.18%. We detected 209 dispersed repeat sequences of 30 bp or more, with the longest of 966 bp, including 120 palindromic repeats and 89 reverse repeats. 43 tandem repeats were identified, ranging from 12 to 51 bases; 18 showed a 100% match rate, the lowest was 78%, and over half exceeded 90% (**Figure 2C**; **Supplementary Tables 2, 3**).

**Figure 3:**
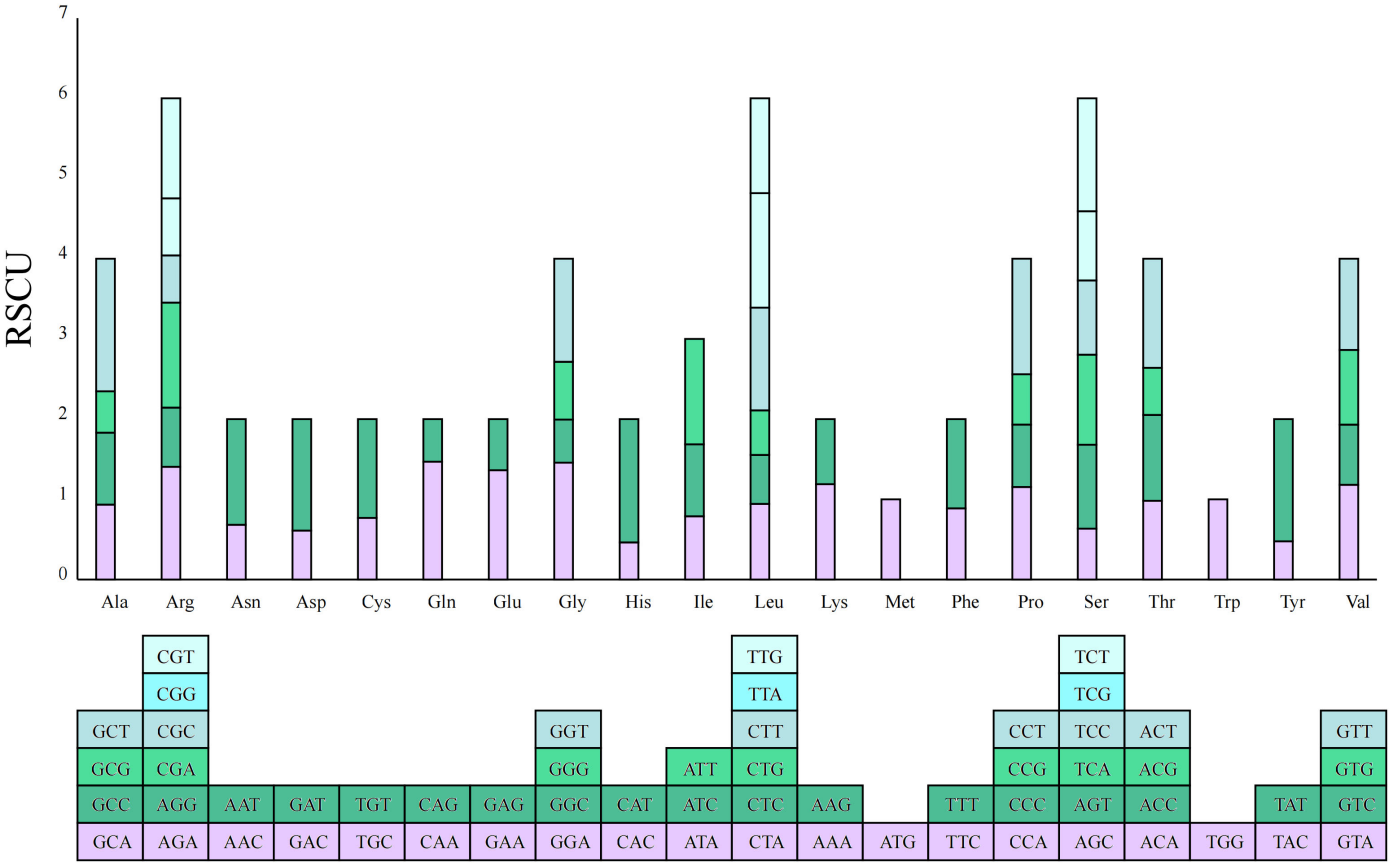
RSCU values for the *G. forrestii* mitochondrial genome. The X-axis represents the amino acids, while the RSCU values reflect the observed frequency of each codon relative to its expected frequency under uniform synonymous codon usage.

A total of 20 types of amino acids (excluding stop codons) and a total of 10,355 codons were found in the mitochondrial genome of *G. forrestii*. The main start codon is AUG, with an RSCU value of 1, while the stop codons are UAA, UGA, and UAG. Each amino acid can be encoded by multiple codons. The three most commonly used amino acids are leucine (Leu), serine (Ser), and arginine (Arg), each with 6 codons. In contrast, tryptophan (Trp) and methionine (Met) each have only 1 codon (**Figure 3**). The RSCU value measures codon usage frequency, with values greater than 1 indicating higher-than-expected usage. In this genome, 12 amino acids have RSCU values greater than 1, including Asp (GAU), Glu (GAA), His (CAU), Ile (AUU), Leu (UUA), Asn (AAU), Gln (CAA), Arg (AGA), Ser (UCU), Thr (ACU), and Tyr (UAU). Additionally, there are 34 amino acids with RSCU values less than 1 (**Supplementary Table 4**).

### 3.3 RNA editing events

**Figure 4:**
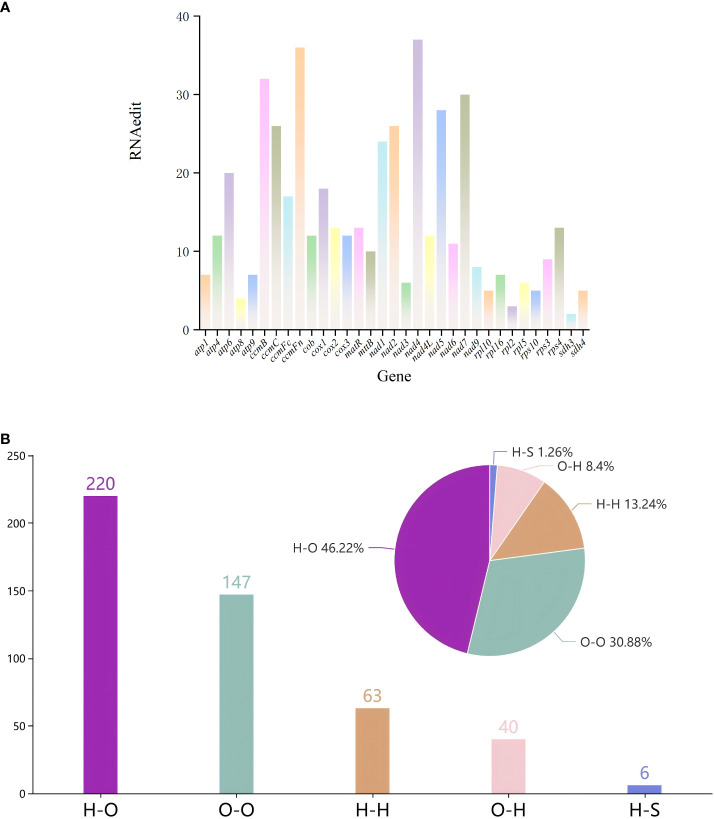
RNA editing events in the mitochondrial genome of *G*. *forrestii.* **(A)** Number of RNA editing sites in all PCGs. **(B)** Types of amino acid substitutions resulting from RNA editing: H-H, Hydrophilic-Hydrophilic; H-O, Hydrophilic-Hydrophobic; H-S, Hydrophilic-Stop; O-H, Hydrophobic-Hydrophilic; and O-O, Hydrophobic-Hydrophobic.

We identified 476 RNA editing sites across 33 protein-coding genes (PCGs) in the mitochondrial genome of *G. forrestii* (**Supplementary Table 5**). Among the 33 PCGs, nine genes have over 20 RNA editing sites: *atp6, ccmB, ccmC, ccmFn, nad1, nad2, nad4, nad5*, and *nad7*. Eleven genes have more than 10 RNA editing sites: *atp4, ccmFc, cob, cox1, cox2, cox3, matR, mttB, nad4L, nad6*, and *rps4*. The remaining 13 genes contain fewer than 10 RNA editing sites (**Figure 4A**).

All the 476 RNA editing sites revealed four major types of editing. Hydrophilic-hydrophilic editing occurred 63 times, accounting for 13.24% of the total. Hydrophilic-hydrophobic editing was the most frequent, occurring 220 times, of 46.22% of the total. This type of editing substitutes a hydrophilic amino acid with a hydrophobic one, for example, ACA (threonine, T) is edited to ATA (isoleucine, I). Hydrophilic-stop editing appeared 6 times, accounting for 1.26% of the total. Hydrophobic-hydrophilic editing was observed 40 times, comprising 8.40% of the total. Hydrophobic-hydrophobic editing occurred 147 times, representing 30.88% of the total (**Figure 4B**; **Supplementary Table 6**).

### 3.4 Collinearity among the mitochondrial genomes

**Figure 5:**
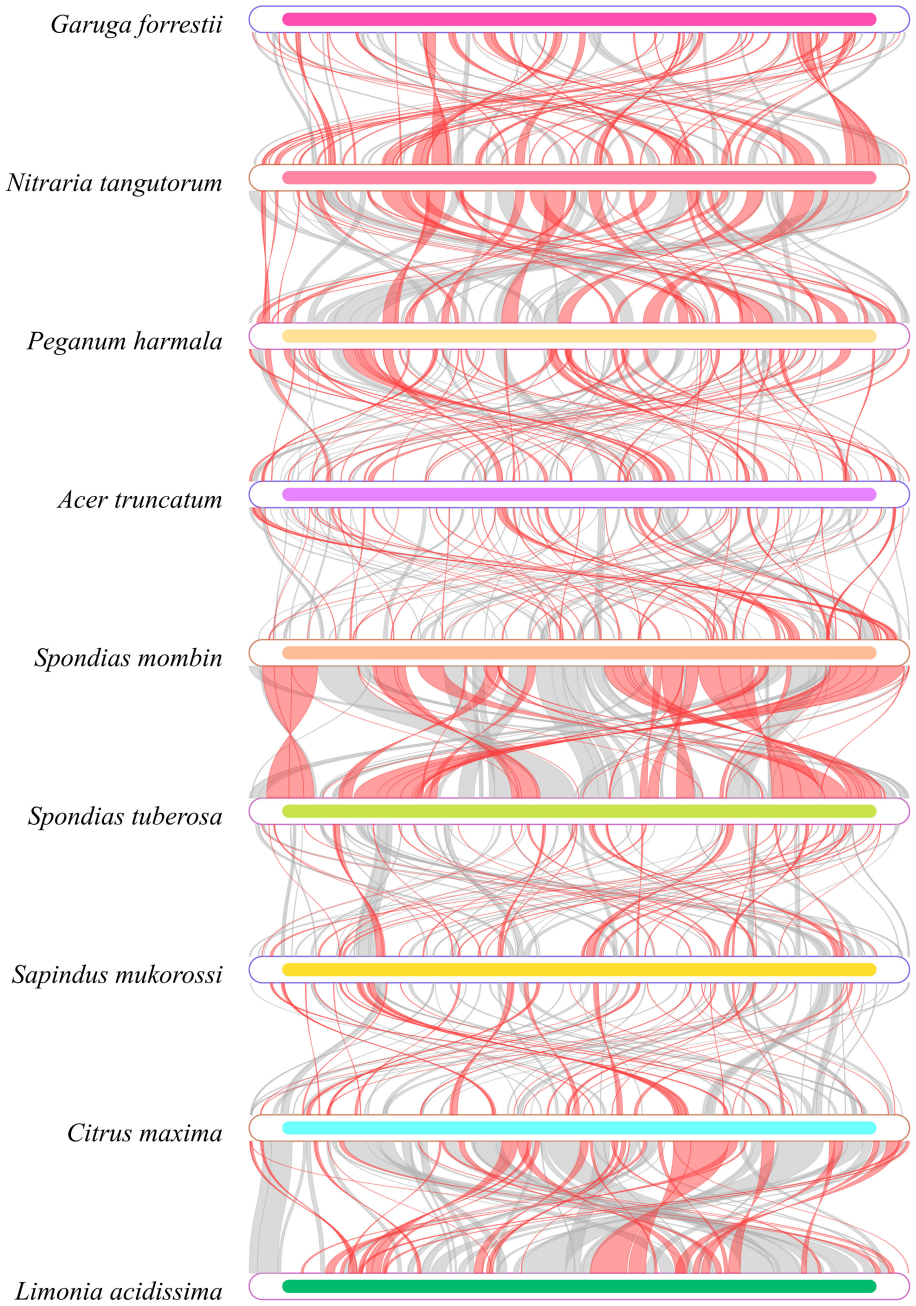
Homology map of the *G. forrestii* genome compared to related species. Boxes represent genomes, with connecting lines showing homologous regions. Red arcs indicate inversions, and gray arcs denote high homology.

In our analysis of the homologous regions within the mitochondrial genome of *G. forrestii* and eight other species, we noted significant differences in the arrangement of these regions. Using *G. forrestii* as a reference, the proportion of homologous sequences in the other species ranged from 25.69% to 39.26%, with lengths varying between 160,667 and 244,984 base pairs (**Supplementary Table 7**). *G. forrestii* exhibits a high degree of similarity with *Spondias tuberosa* and *Spondias mombin*, suggesting a stronger genetic relationship between these species. Conversely, when examining each species individually, *Nitraria tangutorum* demonstrated an impressive similarity of 46.73% to *G. forrestii*, indicating a robust differentiation and close evolutionary relationship. Other species such as *Citrus maxima* and *Limonia acidissima* displayed relatively low levels of similarity, highlighting their more distant genetic connections to *G. forrestii* (**Figure 5**).

### 3.5 Homologous fragments

**Figure 6:**
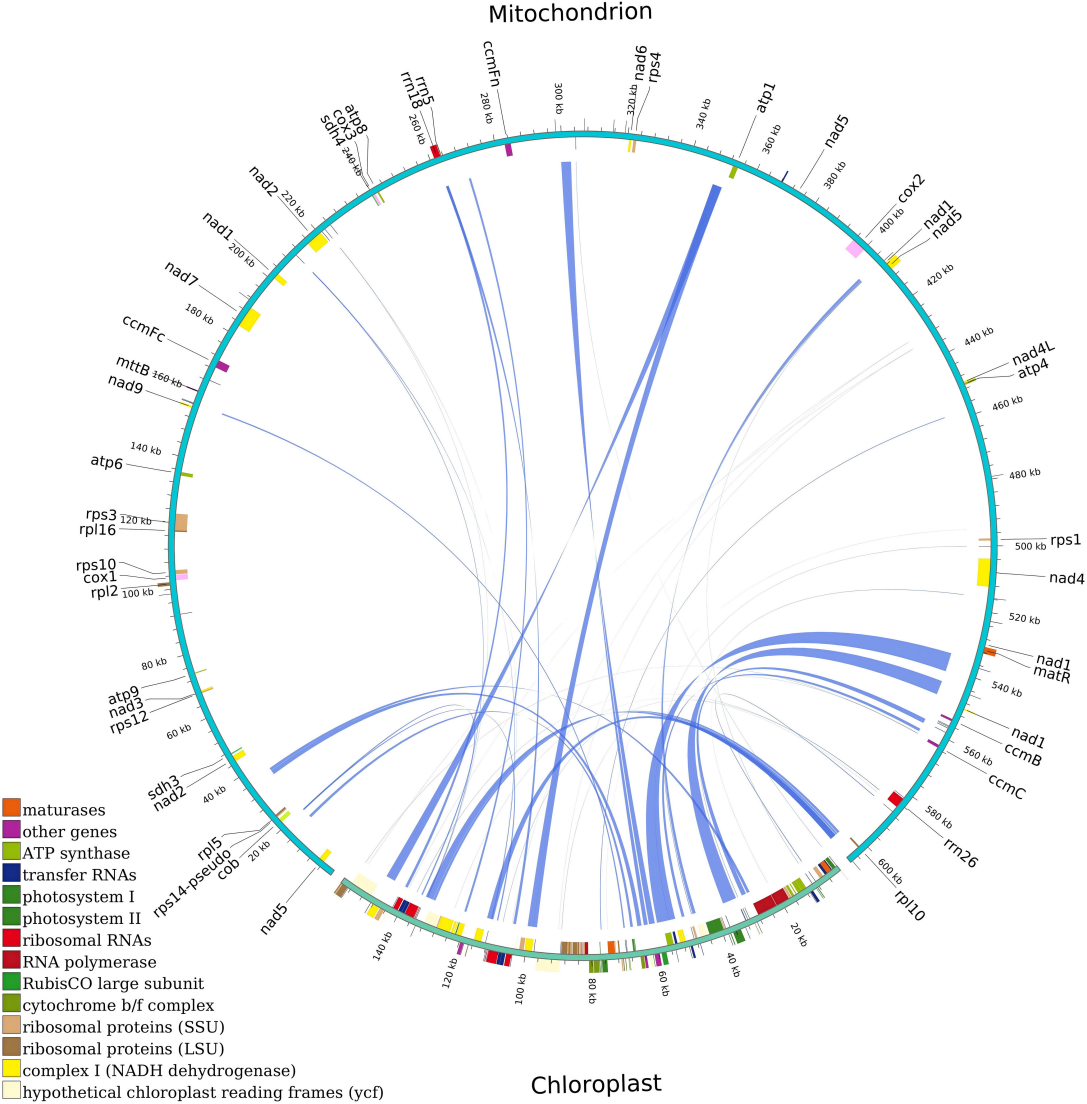
Homologous fragments of chloroplast and mitochondrial gene sequences in *G. forrestii*. Chloroplast and mitochondrial sequences are marked, with homologous genes from the same complex shown in the same color. Connecting lines represent homologous sequences.

In the mitochondrial and chloroplast genomes of *G. forrestii*, we identified 49 homologous fragments with a total length of 37,937 bp (**Supplementary Table 8**). The proportion of homologous sequences in the chloroplast genome was 22.93%. Genes completely located within these homologous sequences include various tRNA genes and functional genes (such as *atpE, psbD, accD*). Genes partially located within the homologous sequences and their proportions include *ndhC* (62.81%), *rbcL* (71.29%). In the mitochondrial genome, the proportion of homologous sequences was 5.05%, with genes completely located in these sequences including tRNA genes (such as *trnM-CAT*, *trnT-GGT*). Genes partially located within the homologous sequences and their proportions include *rrn18* (43.72%) and *trnN-GTT* (98.61%) (**Figure 6**; **Supplementary Table 9**).

### 3.6 Nucleotide diversity and selection pressure (Ka/Ks) analysis

**Figure 7:**
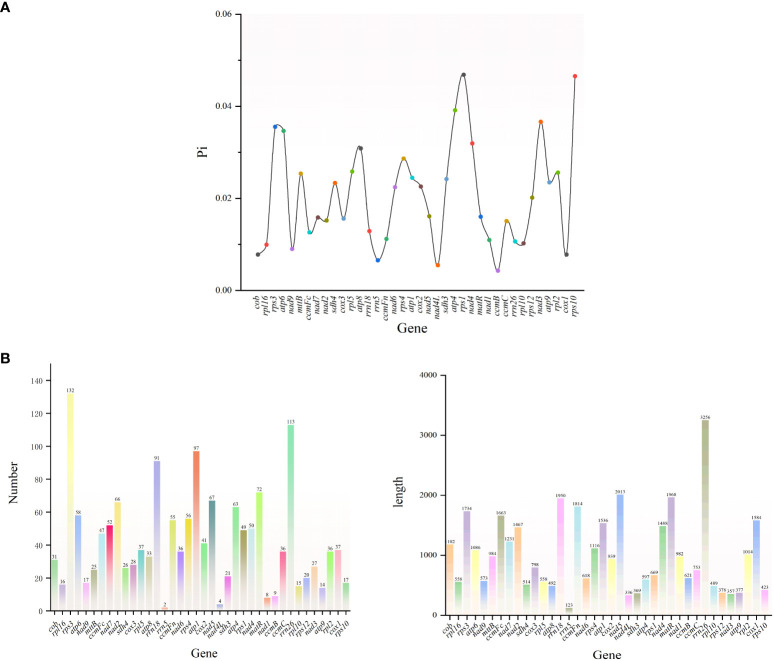
**(A)** Nucleotide diversity (Pi) of the mitochondrial genome of *G. forrestii*. **(B)** Genes of the mitochondrial genome of *G. forrestii*, showing the number and relative lengths of each gene.

The variable region lengths of *G. forrestii* ranged from 123 to 3256 bp, and the total number of mutations varied from 2 to 132. Most genes were found to have Pi values below 0.040, with the highest recorded in *rps1* (0.046) and the lowest in *nad4L* (0.005). A total of 21 genes were identified with mutations exceeding the average of 36, with *rps3* having the highest count of 132, and *rrn5* the lowest count of 2. 15 genes were found to have lengths exceeding the average of 1075 bp, with *rrn26* being the longest (3256 bp) and *rrn5* the shortest (123 bp) See (**Figures 7A, B**).

**Figure 8:**
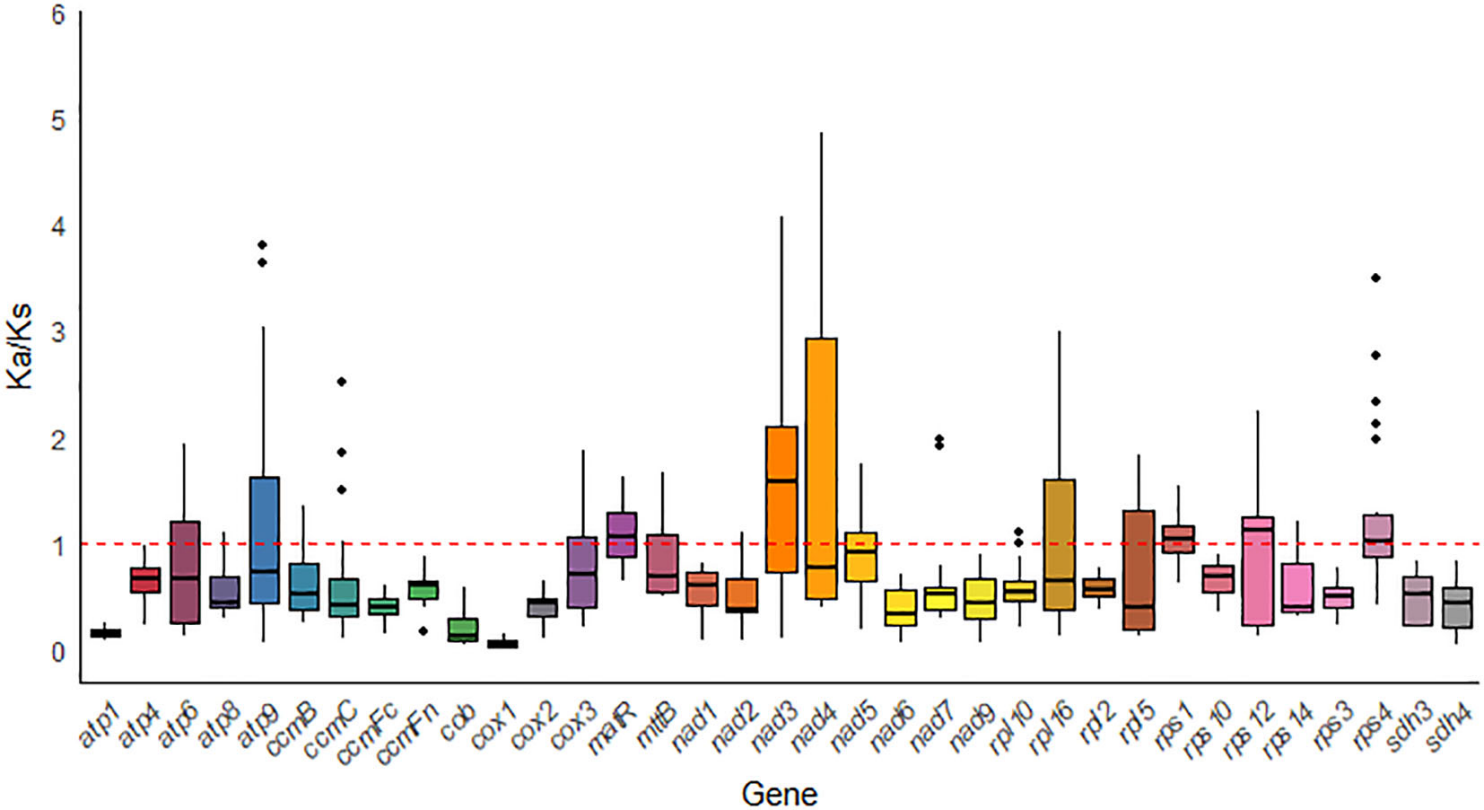
Box plot of pairwise Ka/Ks ratios for mitochondrial genes across eight Sapindales species.

To assess the evolutionary selective pressures on mitochondrial protein-coding genes (PCGs) in closely related species, we calculated the ratio of nonsynonymous substitutions (Ka) to synonymous substitutions (Ks) (Ka/Ks). In the analysis of 35 PCGs from *G. forrestii* and 8 other Burseraceae species, most genes showed evidence of purifying selection (Ka/Ks < 1), suggesting that conservative evolutionary pressures were dominant. However, several genes, such as *matR*, *nad3*, *rps1*, *rps12*, and *rps4*, exhibited Ka/Ks ratios greater than 1, indicating their involvement in specific adaptive evolutionary processes (see **Figure 8**). Notably, the *cox1* gene displayed the lowest Ka/Ks value (0.067) across all species examined, indicating that it has experienced strong purifying selection and remains highly conserved throughout evolution.

### 3.7 Phylogenomic of mitochondrial

**Figure 9:**
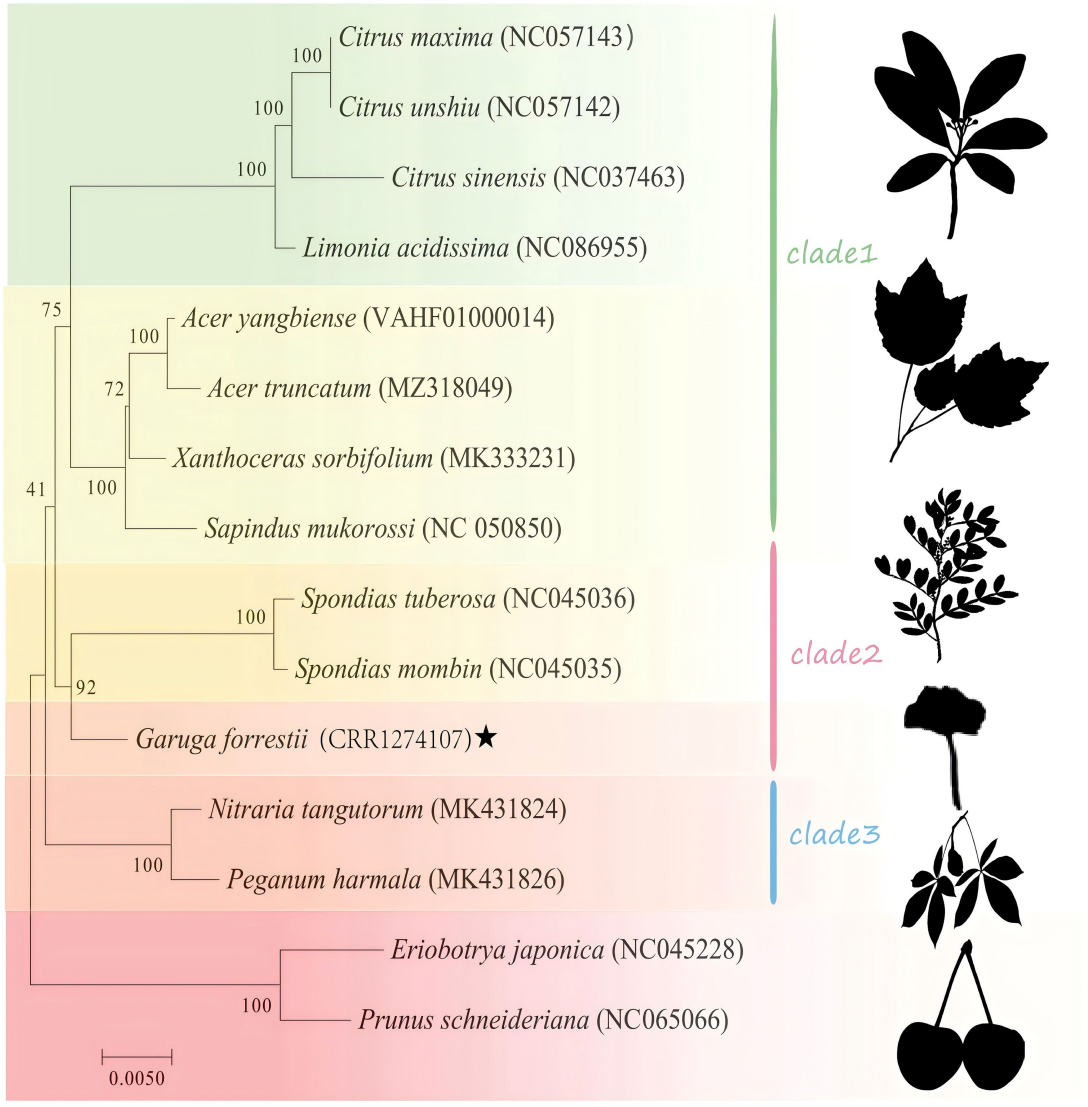
Phylogenetic tree of mitochondrial genes for 15 species, with bootstrap support values shown as percentages from 1,000 replicates. Species within the same family are indicated by the same color block, with *G. forrestii* highlighted by ★.

Phylogenetic analysis was performed for 15 species in the Sapindales order, with Rosaceae as the outgroup (**Supplementary Table 1**). As shown in **Figure 9**, *Nitraria tangutorum*, *Peganum harmala* were highly supported to cluster at the basal position. Burseraceae (*G. forrestii*) was determined to be a monophyletic group with a high bootstrap support of 92, indicating a close relationship with Anacardiaceae (*Spondias mombin* and *Spondias tuberosa*). Four species in the Rutaceae family (*Citrus maxima*, *Citrus unshiu*, *Citrus sinensis*, and *Limonia acidissima*) all had bootstrap support of 100. The mitochondrial phylogenetic genome tree was consistent with the topology of APG IV.

## 4 Discussion

### 4.1 Features of mitochondrial genome of *G. forrestii*

Mitochondria are the energy-producing organelles in plants, essential for supporting vital life processes. Plant mitochondria genomes that are more complex than those of animals, characterized by extensive variation in genome size, sequence organization, repetitive content, and highly conserved coding sequences (Zhou et al., 2022). Due to high recombination frequency, plant mitochondrial genomes exhibit dynamic structures with various conformations within mitochondria, including multiple forms such as master circles, subgenomic circles, and linear molecules. For example, cucumber (*Cucumis sativus*) possesses three circular molecules (Alverson et al., 2011), whereas rice (*Oryza sativa*) displays a linear structure (Notsu et al., 2002). In this study, the mitochondrial genome of *G. forrestii* shows the typical circular structure found in terrestrial plants, with a length of 606,853 bp and a GC content of 45%, similar to *Gleditsia sinensis* (594,121 bp; 45.3%) (Yang et al., 2021). The plant mitochondrial genome, as a fascinating molecular entity, has sparked widespread research interest regarding how the high variability of its non-coding regions and structural mutations are generated, repaired, retained, and fixed (Wang et al., 2024a). The size of plant mitochondrial genomes varies significantly among different species, with differences reaching up to 200-fold (Wang et al., 2024a). Generally, plant mitochondrial genomes contain a large amount of non-coding DNA sequences, which occupy a substantial proportion of the genome and contribute to its overall size (Shidhi et al., 2021). Among reported plant mitochondrial genomes, the longest is 11.3 Mb (*Silene conica*) (Gray et al., 2012), while the shortest is 0.66 Mb (*Viscum scurruloideum*) (Skippington et al., 2015). Compared to the mitochondrial genome lengths of related plants in the Sapindales available in the NCBI database (see **Supplementary Table 11**), the mitochondrial genome of *G. forrestii* (606,853 bp) is of moderate size. Furthermore, *G. forrestii* contains 35 protein-coding genes (PCGs), a number comparable to that of other angiosperms, such as *Cymbidium ensifolium* (35 PCGs) (Shen et al., 2024).

### 4.2 Comparative analysis of the mitochondrial genome of *G. forrestii*

Repeat sequences are commonly found in mitochondrial genomes, including tandem repeats, short repeats, and large repeats (Gualberto et al., 2014; Guo et al., 2017). Previous studies have demonstrated that repetitive sequences are crucial for gene expression and regulation (Shapiro and von Sternberg, 2005). They are also a major driving force for gene diversification and evolution (Lynch and Conery, 2000). We identified 120 forward repeats and 89 reverse repeats, but no complementary or palindromic repeats were found. The existence of these repetitive sequences may play a key role in the structural complexity and evolutionary dynamics of the genome. Over time, these sequences may dynamically alter the structure and conformation of the mitochondrial genome, driving its rearrangement and evolution. We observed duplications in several genes within the mitochondrial genome of *G. forrestii*, a phenomenon also documented in other species (Yang et al., 2022; Lu et al., 2023). These duplications are thought to arise from horizontal gene transfer (HGT) or mtDNA recombination during evolutionary processes (Yu et al., 2023; Zeng et al., 2024). Previous studies have highlighted that mitochondrial repetitive sequences harbor substantial genetic information and play a critical role in intramolecular recombination (Xu et al., 2013). Notably, the *trnM-CAT* gene appears four times in the mitochondrial genome of *G. forrestii*, a pattern also observed in *Hippophae tibetana* (Zeng et al., 2024), suggesting that this gene may play an important role in the biology of *G. forrestii*.

RNA editing occurs in the post-transcripe protein folding, which is associated with a decrease in the overall stability of protein structures (Capella-Gutiérrez et al., 2009; Bi et al., 2019). Among the four major editing types internal process of chloroplast (cp) and mitochondrial (mt) genomes in higher plants and can alter the genetic information at the mRNA level, enabling more efficient protein folding (Bi et al., 2016). In our study, we predicted 476 RNA editing sites in the *G. forrestii* mitochondrial genome, less than that of *Oryza sativa* (491) (Notsu et al., 2002), more than that of *Arabidopsis thaliana* (441) (Unseld et al., 1997). We also conducted a search on Google Scholar, retrieving 30 results (**Supplementary Table 12**). The highest RNA editing events count was 2023, while the lowest was 93, most of values ranging from 400-600, 24 of all 30 reports, 80%. Our results fall within this range, further corroborating the relevance and consistency of our findings. RNA editing recognition is crucial for understanding the mitochondrial genome of *G. forrestii*. Since RNA editing can alter amino acids, it often changes their physical and chemical properties, thus affecting protein function. In *G. forrestii*, we found that hydrophilic-hydrophobic and hydrophobic-hydrophobic edits were the most common, accounting for 77.10%. This supports the connection between amino acid hydrophobicity and protein folding, as well as the formation of secondary structures (Li et al., 2016). Nearly 50.00% of the sites are located at the second codon position, with more than half of the amino acids showing hydrophobicity changes (Benson, 1999). The usage frequency (RSCU) of Ala (1.6528 > 1) (Sharp et al., 1986) is significantly higher than that of other amino acids, indicating that it may play a more important role in protein synthesis (Deb et al., 2021). Compared to the first and second codon positions, the third codon position shows a strong A/T bias, containing more compact information sites (Xue et al., 2022), which is commonly observed in the mitochondrial genomes of other plant species (Shidhi et al., 2021). This is considered a result of the long-term evolutionary process by which plants adapt to their environment (Romero, 2000).

Ka/Ks analysis and the comparison of genomic features with other plant mitochondrial genomes help to provide a comprehensive understanding of plant mitochondrial evolution (Ye et al., 2017). Most of the protein-coding genes (PCGs) in *G. forrestii* show evidence of purifying selection (Ka/Ks < 1) during evolution, indicating that the PCGs in the mitochondrial genome are relatively conserved. Consistent with the evolutionary patterns observed in most angiosperms, most PCGs have undergone neutral and negative selection (Yang and Nielsen, 2000; Bi et al., 2016; Cheng et al., 2021). However, genes such as *matR*, *nad3*, *rps1*, *rps12*, and *rps4* have undergone positive selection during evolution. Positive selection pressure has also been reported in other plant species for genes like *matR*, *nad6*, *ccmF*, *ccmB*, *mttB* during evolution (Cheng et al., 2021a; Ni et al., 2022; Ke et al., 2023a; Xia et al., 2023). A low nuclear-tide diversity (Pi < 0.04) indicated limited genetic variation in mitochondrial genome of *G. forrestii*.

### 4.3 Homology and phylogeny of the *G. forrestii* mitochondrial genome

Homologous sequence analysis plays a crucial role in phylogenetic tree construction, providing reliable data support for understanding gene evolutionary history and evolutionary relationships between species (Kapli et al., 2020). Through homology analysis, it is possible to reveal complex ancestral evolutionary trends and independent gene loss patterns (Sjölander, 2004). By combining the genetic distance matrix and homology data of *G. forrestii*, we found a positive correlation between the homologous regions (**Figure 5**), with lengths ranging from 160,667 to 244,984. *G. forrestii* shows a high degree of similarity with *Nitraria tangutorum* and *Peganum harmala*, suggesting a close relationship between them. This is consistent with their positions in the APG IV angiosperm phylogenetic tree (Group et al., 2016).

The mitochondrial genome of higher plants evolves slowly and has a low mutation rate (Gray et al., 2012; Wang et al., 2024b), making it an ideal tool for phylogenetic studies (Liu et al., 2013). Mitochondrial DNA sequences serve as valuable markers in phylogenetic reconstruction, as described in other taxa by Hiesel et al. (1994) and Burger et al. (2003). In this study, phylogenetic analysis was conducted on the mitochondrial genome of *G. forrestii* and the mitochondrial genomes of 14 other plant species based on information obtained from the mitochondrial genome. The evolutionary relationships among these species align with the topology of the phylogenetic tree, indicating consistency between traditional taxonomy and molecular classification. However, the node where Clade 1 and Clade 2 (**Figure 9**) was diverged, is poorly supported by ultra-bootstrap tests (only 41%). To test the effects of horizontal gene transfer (HGT) on phylogenetic reconstruction, we performed a BLAST analysis on highly variable loci, but found no evidence of HGT signals. All the high informative segments are all from most related taxa (**Supplementary Tables 14**; **Supplementary Figures 2–14**). After filtering for highly variable loci, we observed that bootstrap probabilities did not improve significantly, increasing by only 5% (see **Supplementary Figure 15**). Substitution saturation may be the primary reason for this lower support, while the distant relationships between species and the incompleteness of the data could also contribute significantly. This highlights the potential of using information obtained from the mitochondrial genome in plant phylogenetic studies. These findings lay a foundation for understanding evolutionary relationships within Burseraceae. However, with few representative mitochondrial genomes available, more sequencing is needed to better resolve the phylogeny and evolution of the family. Expanding sequenced lineages will deepen our understanding of genome evolution (Wang et al., 2024a).

## Data Availability

The raw sequence data reported in this paper have been deposited in the Genome Sequence Archive (Genomics, Proteomics & Bioinformatics 2021) in National Genomics Data Center (Nucleic Acids Res 2022), China National Center for Bioinformation / Beijing Institute of Genomics, Chinese Academy of Sciences (GSA: CRA021850) that are publicly accessible at https://ngdc.cncb.ac.cn/gsa.
