# Supplementary figures and images for: Features and evolutionary adaptations of the mitochondrial genome of Garuga forrestii W. W. Sm
Source: Front Plant Sci. 2025 Jan 20;15:1509669. doi: 10.3389/fpls.2024.1509669 (PMC11788303; doi:10.3389/fpls.2024.1509669)

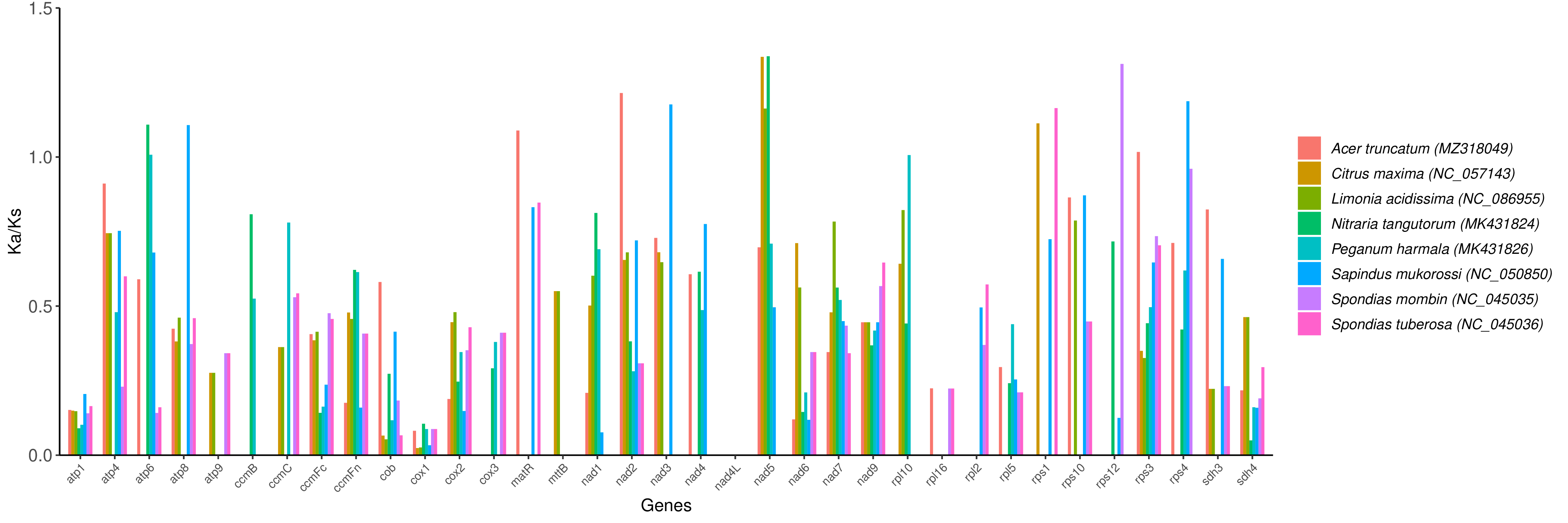

Supplement: Supplementary file 2 [file DataSheet2.zip › 补充图/Supplementary Fig 1.png]

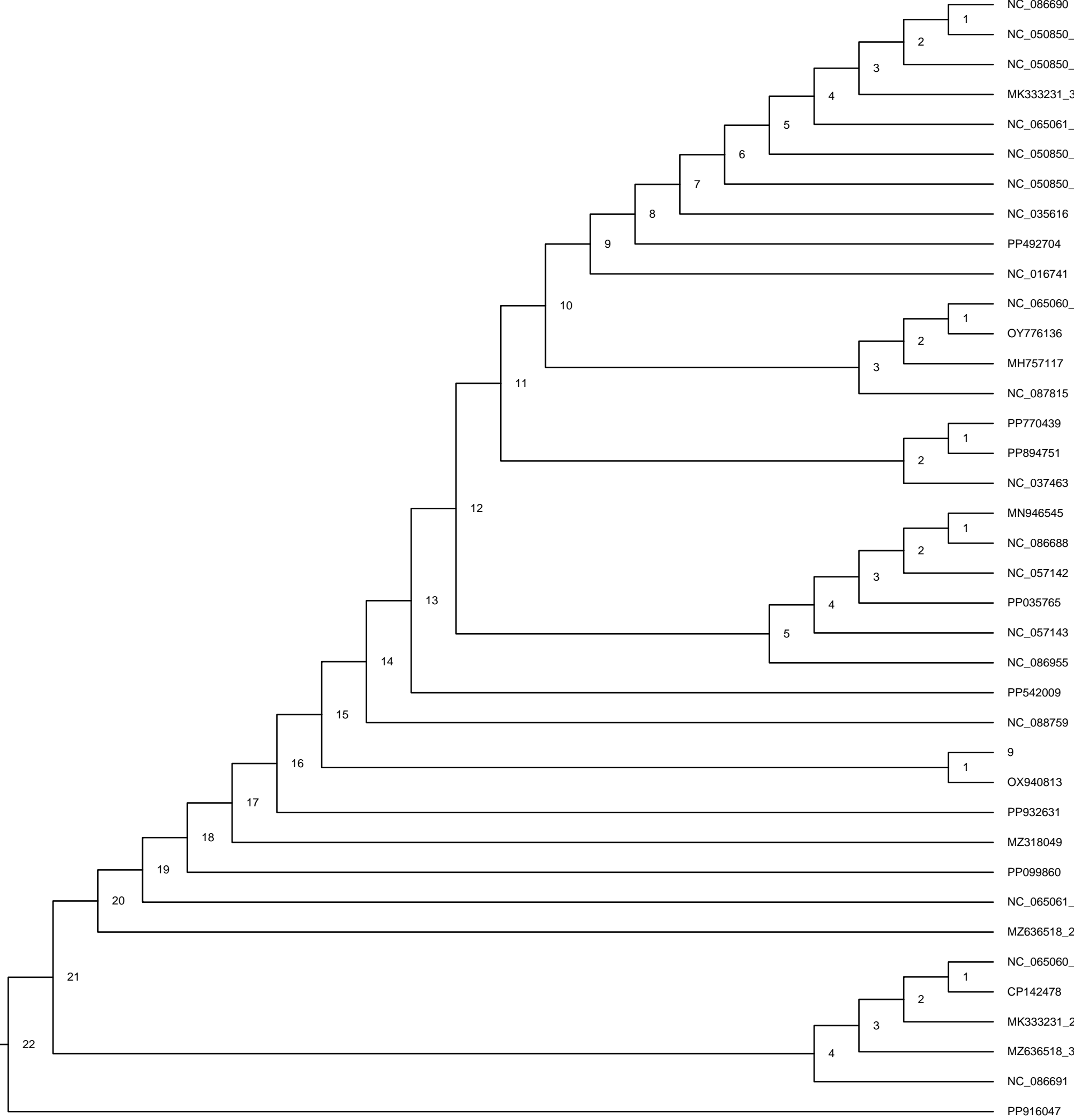

0.2

Supplement: Supplementary file 2 [file DataSheet2.zip › 补充图/Supplementary Fig 10.pdf]

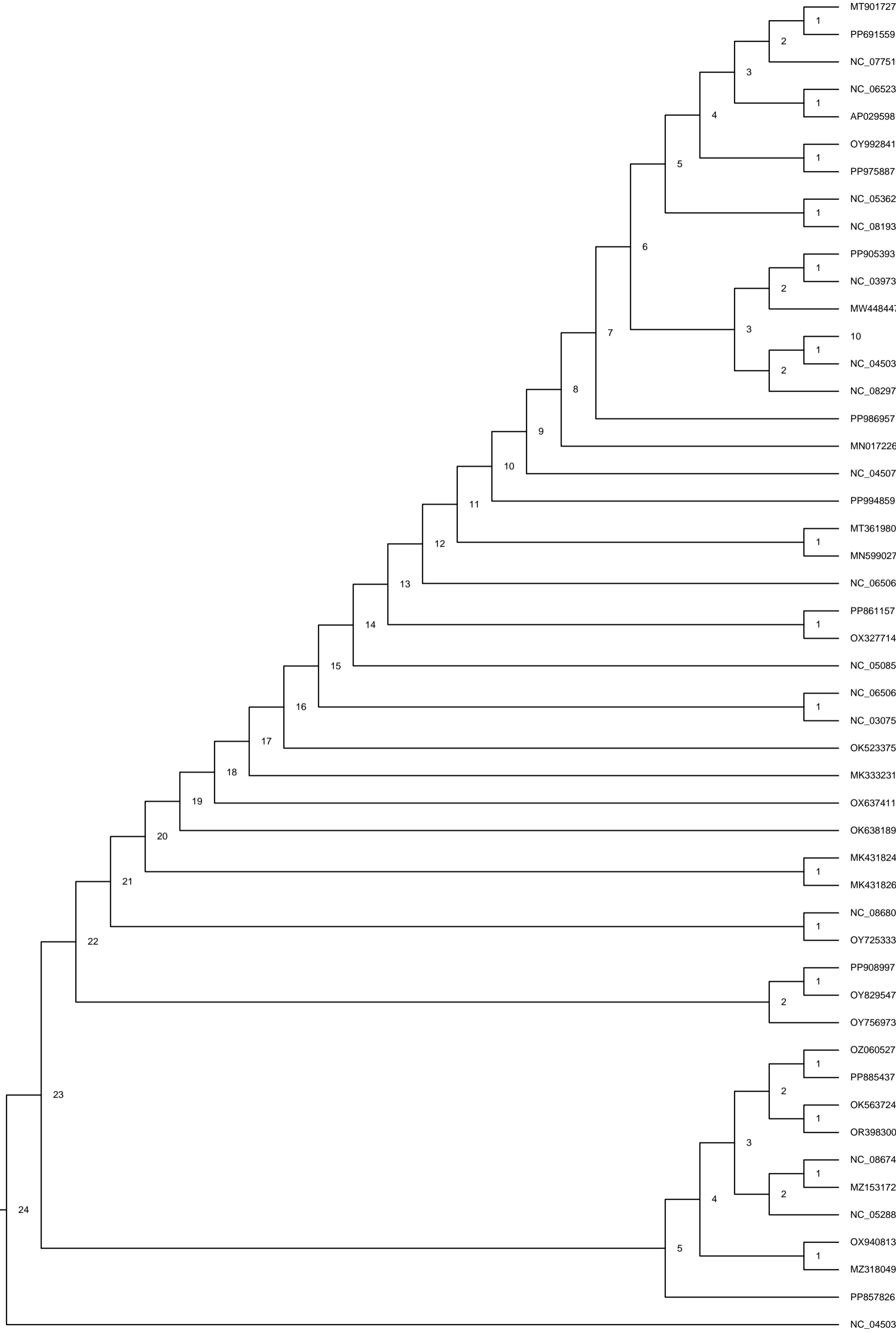

Supplement: Supplementary file 2 [file DataSheet2.zip › 补充图/Supplementary Fig 11.pdf]

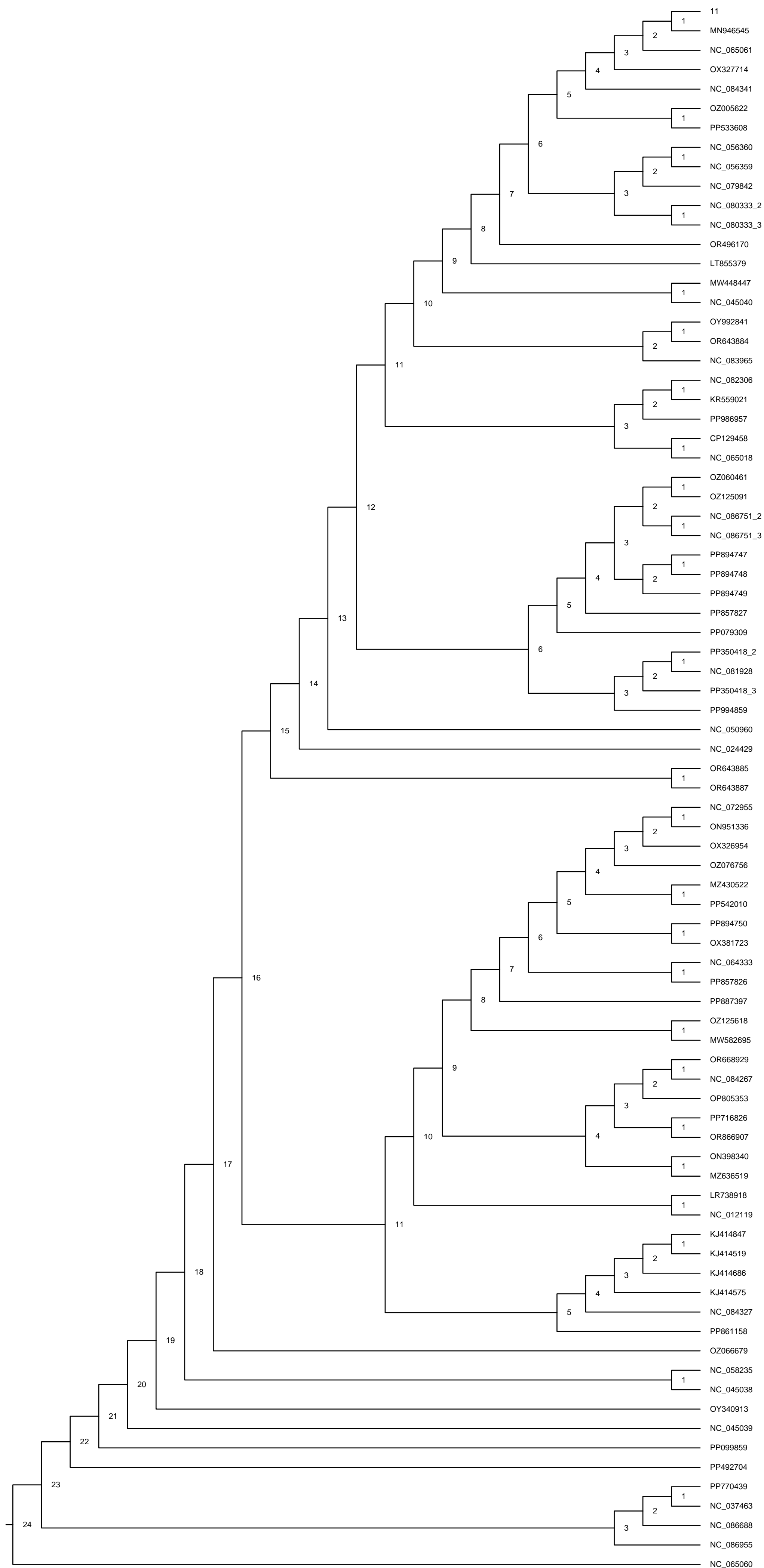

Supplement: Supplementary file 2 [file DataSheet2.zip › 补充图/Supplementary Fig 12.pdf]

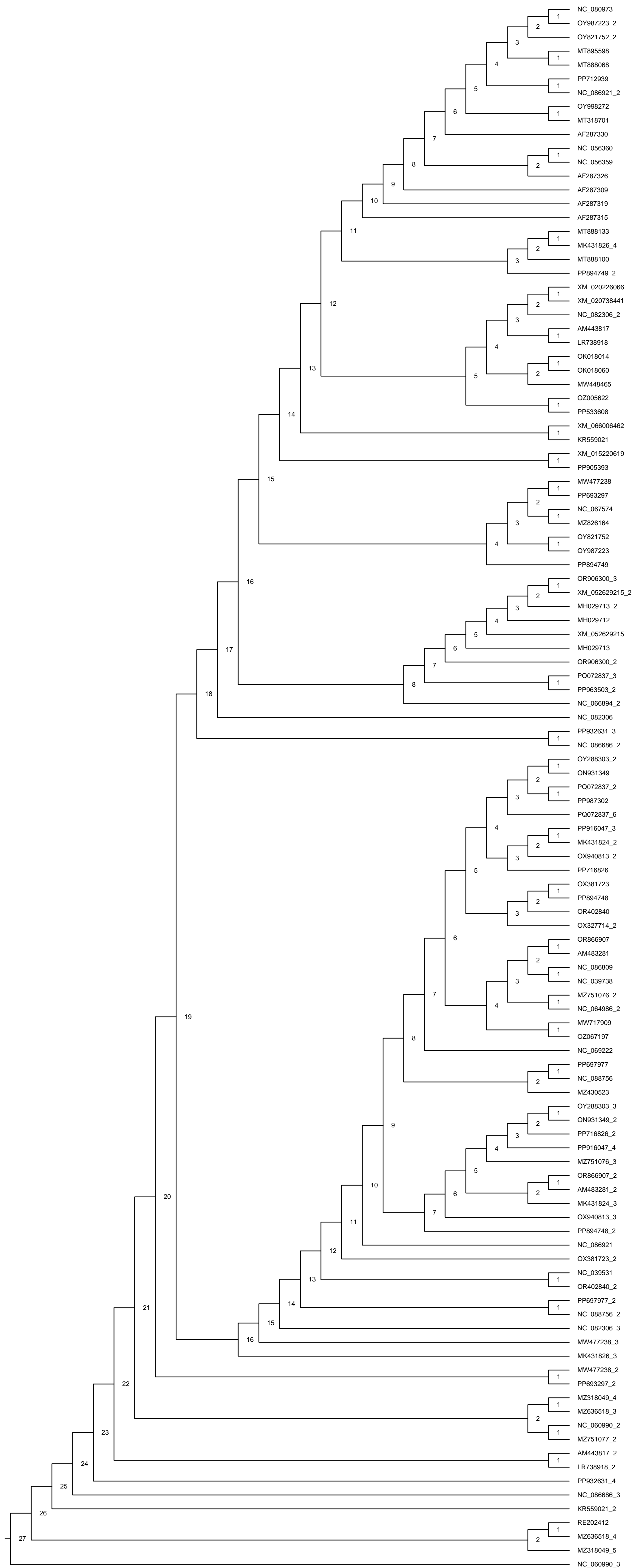

0.5

Supplement: Supplementary file 2 [file DataSheet2.zip › 补充图/Supplementary Fig 13.pdf]

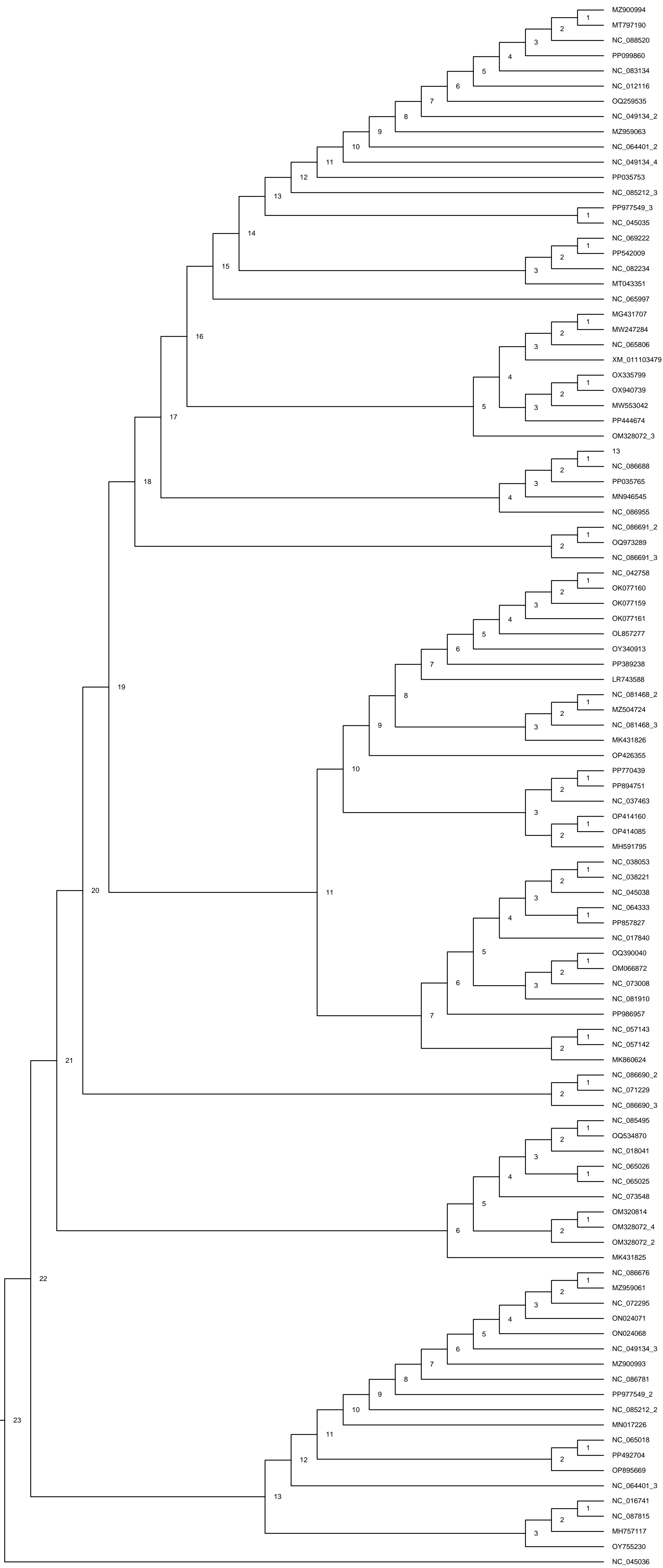

Supplement: Supplementary file 2 [file DataSheet2.zip › 补充图/Supplementary Fig 14.pdf]

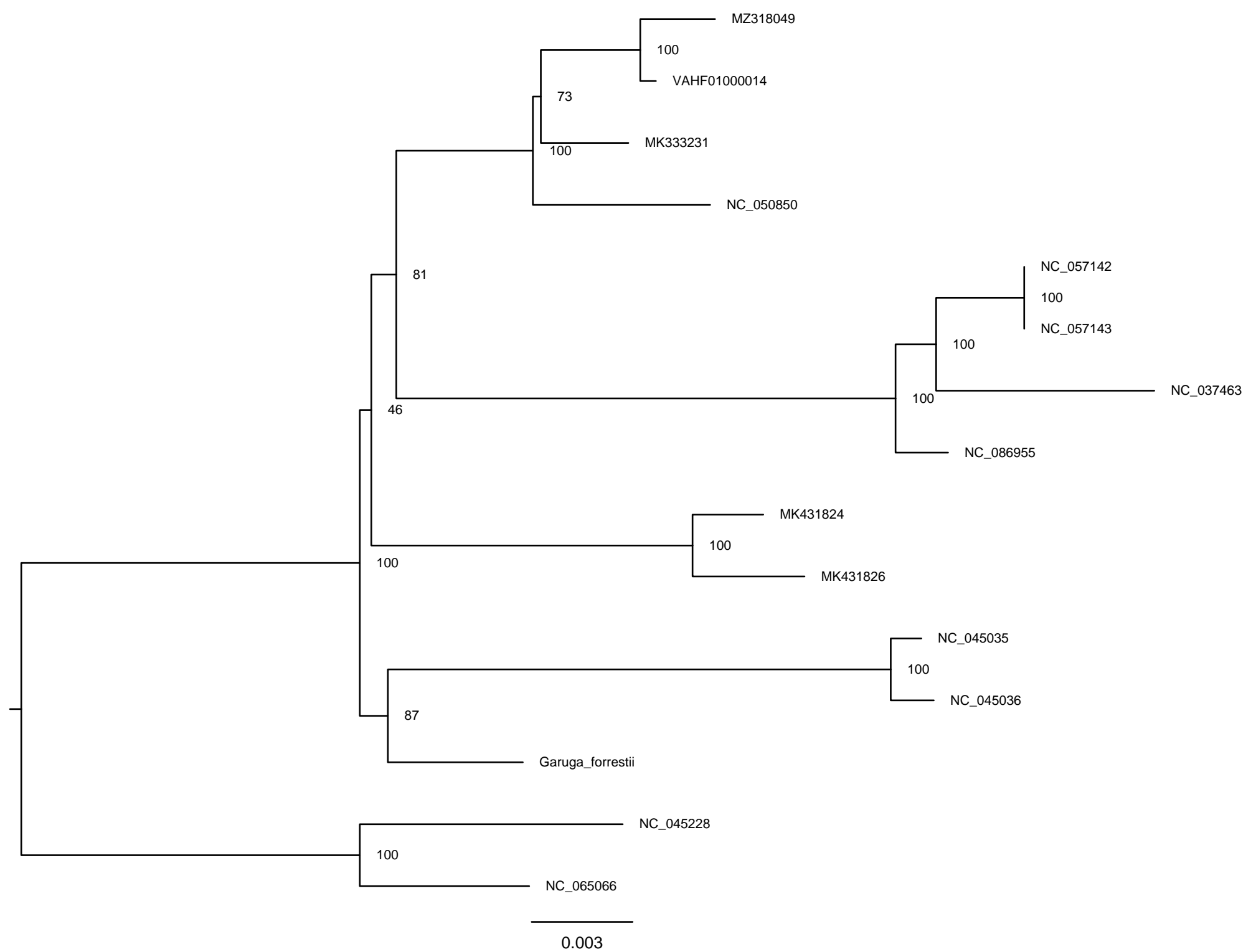

Supplement: Supplementary file 2 [file DataSheet2.zip › 补充图/Supplementary Fig 15.pdf]

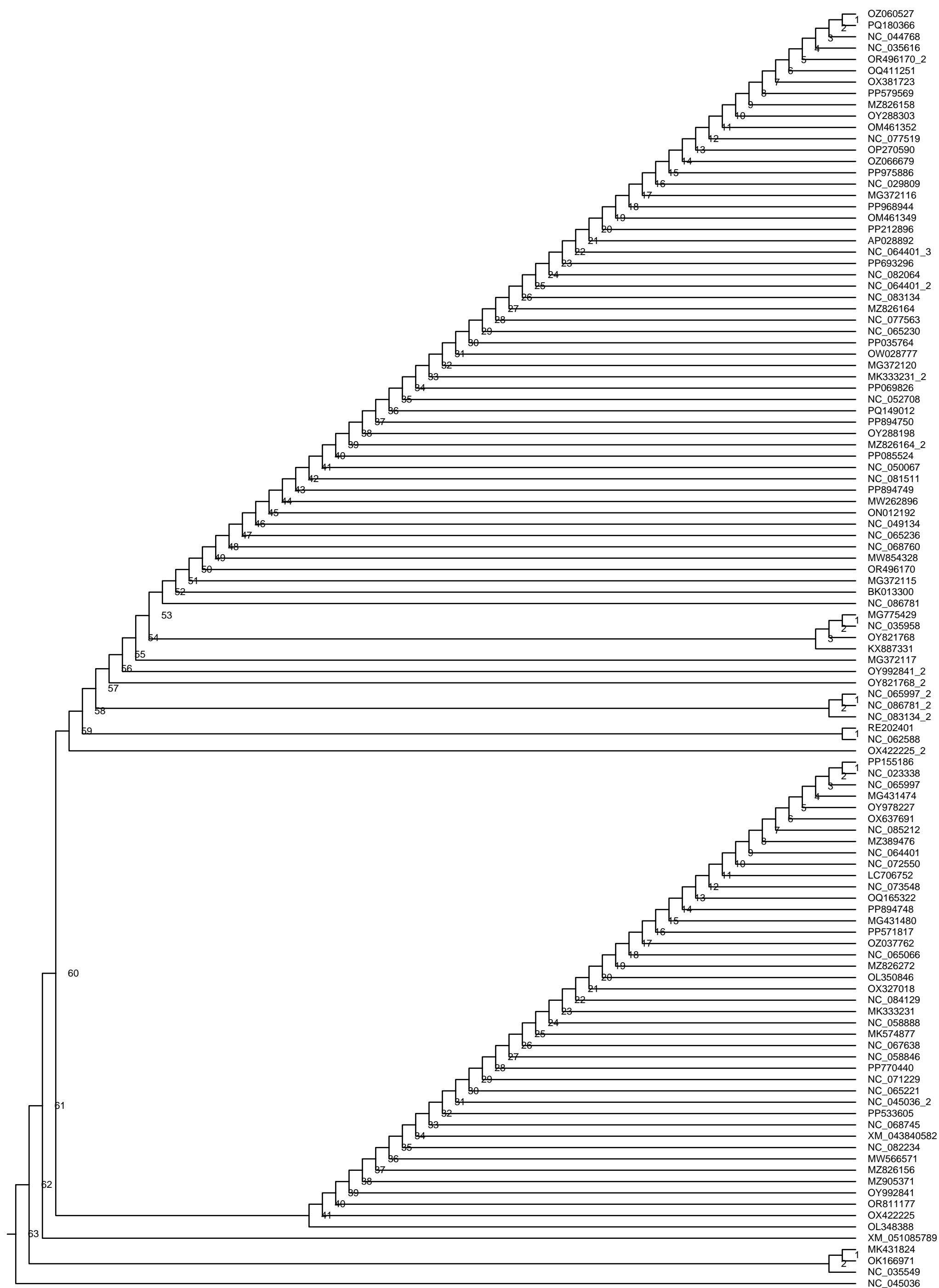

0.08

Supplement: Supplementary file 2 [file DataSheet2.zip › 补充图/Supplementary Fig 2.pdf]

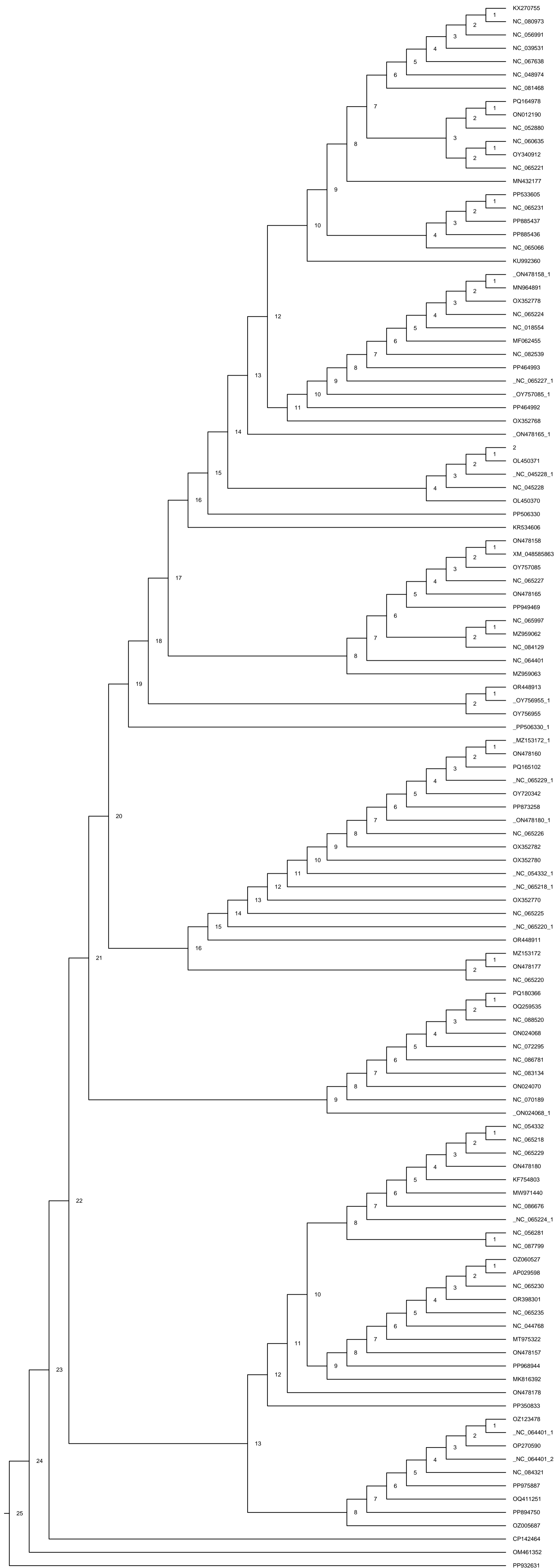

Supplement: Supplementary file 2 [file DataSheet2.zip › 补充图/Supplementary Fig 3.pdf]

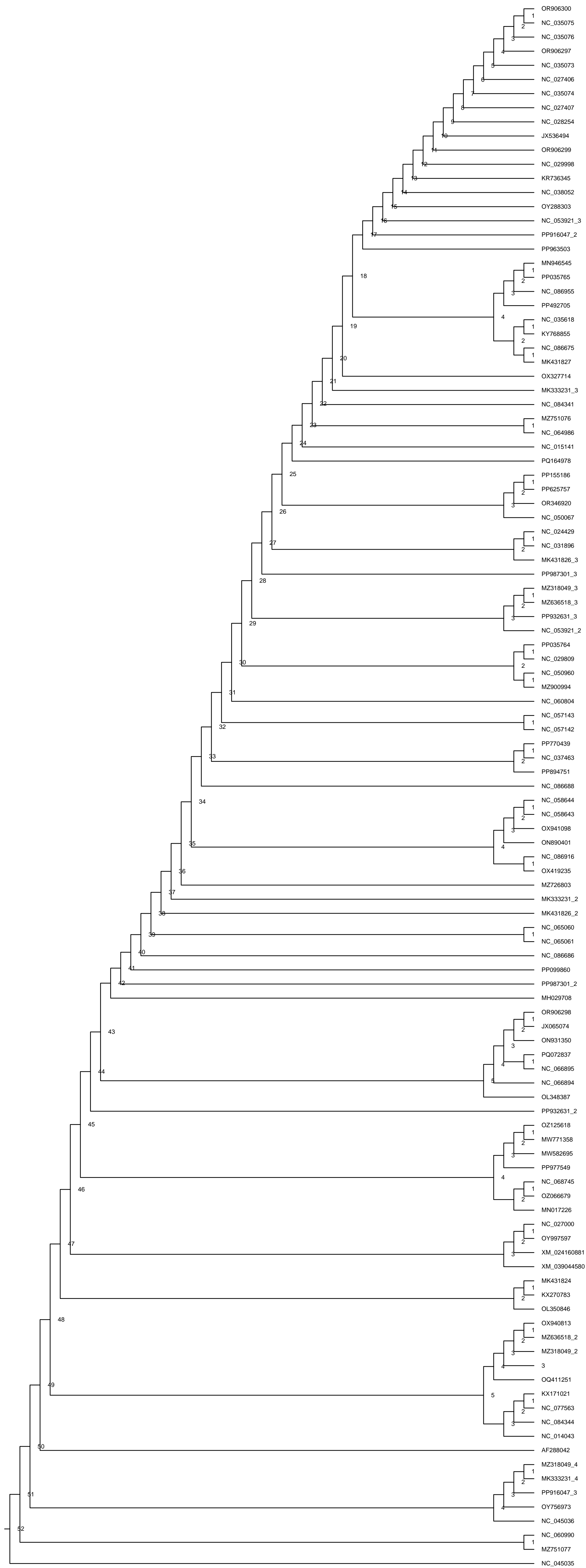

Supplement: Supplementary file 2 [file DataSheet2.zip › 补充图/Supplementary Fig 4.pdf]

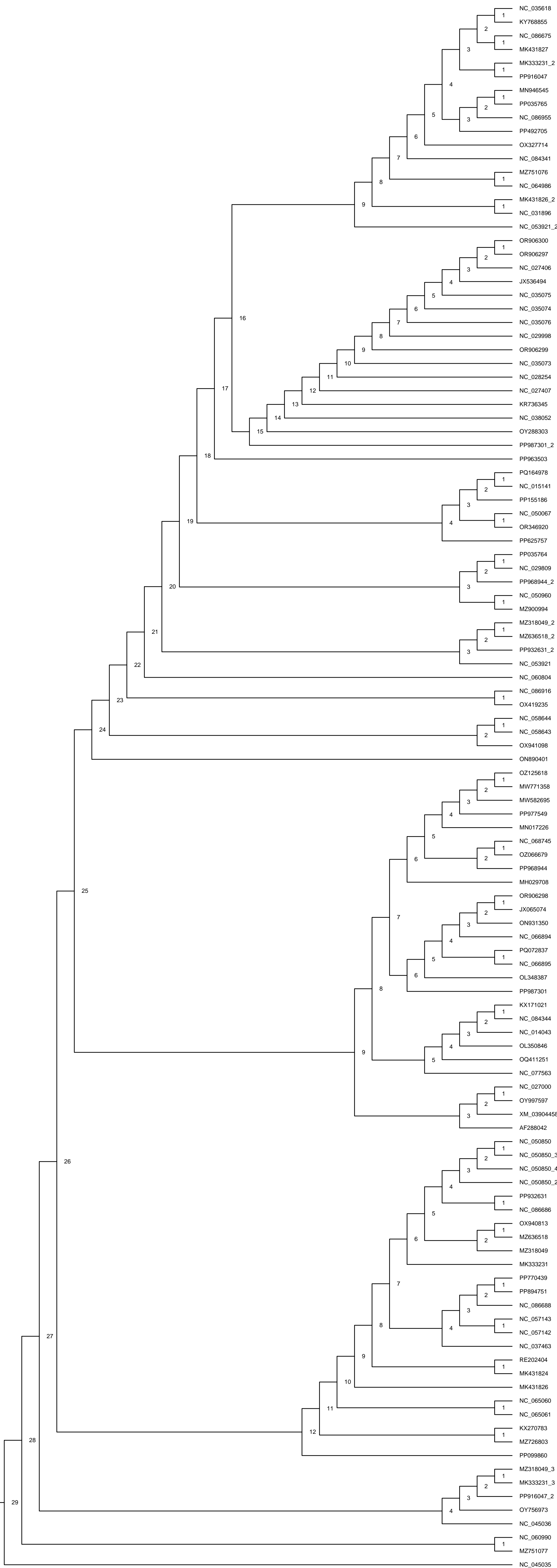

Supplement: Supplementary file 2 [file DataSheet2.zip › 补充图/Supplementary Fig 5.pdf]

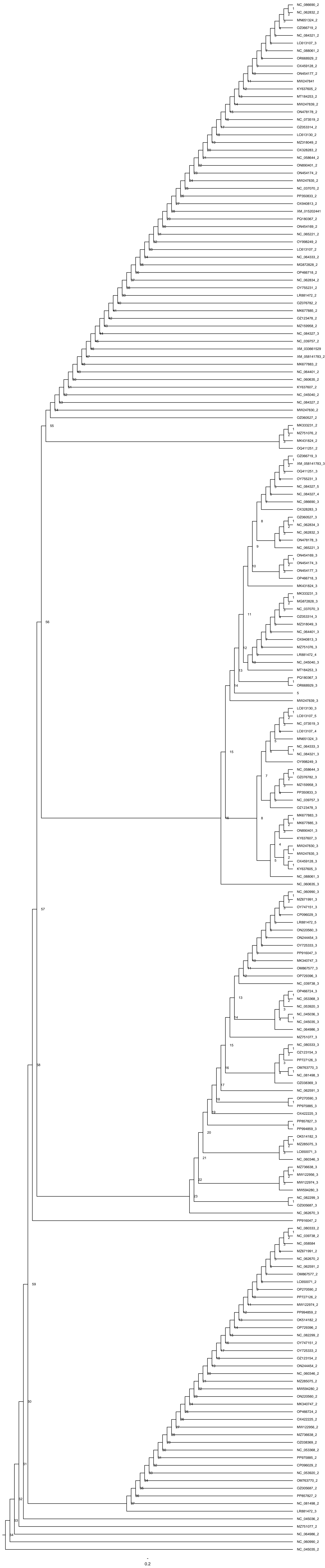

Supplement: Supplementary file 2 [file DataSheet2.zip › 补充图/Supplementary Fig 6.pdf]

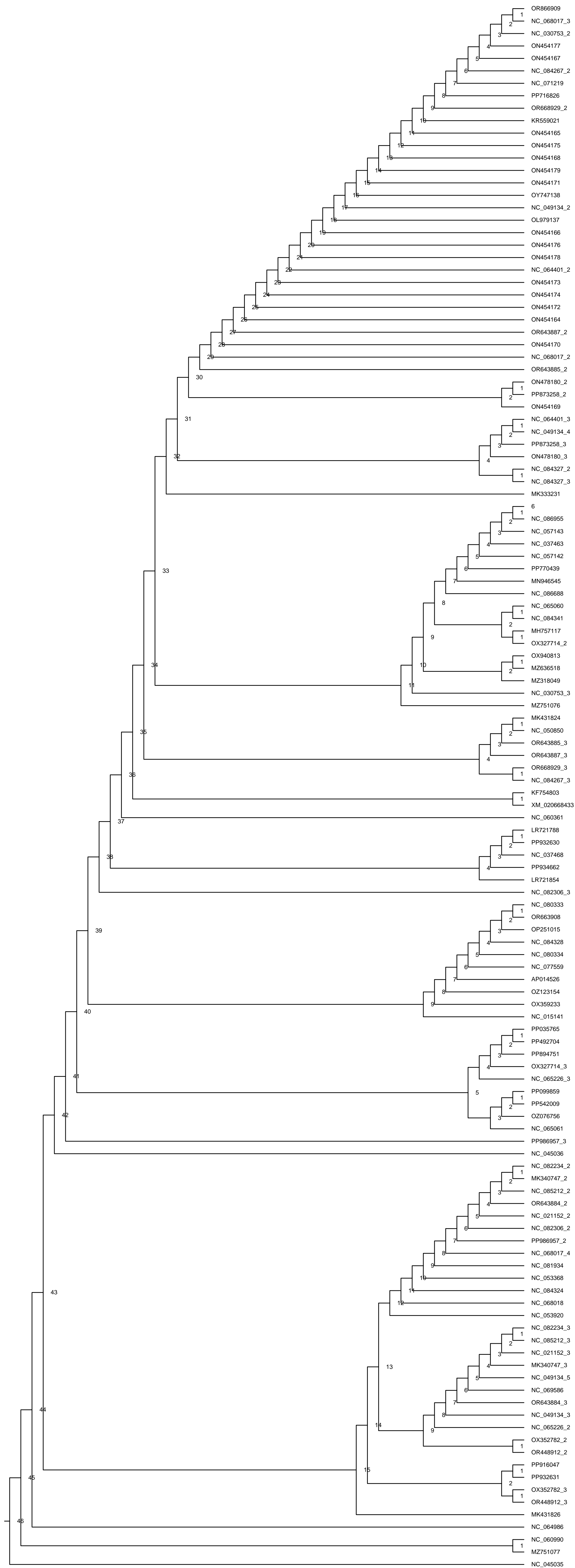

Supplement: Supplementary file 2 [file DataSheet2.zip › 补充图/Supplementary Fig 7.pdf]

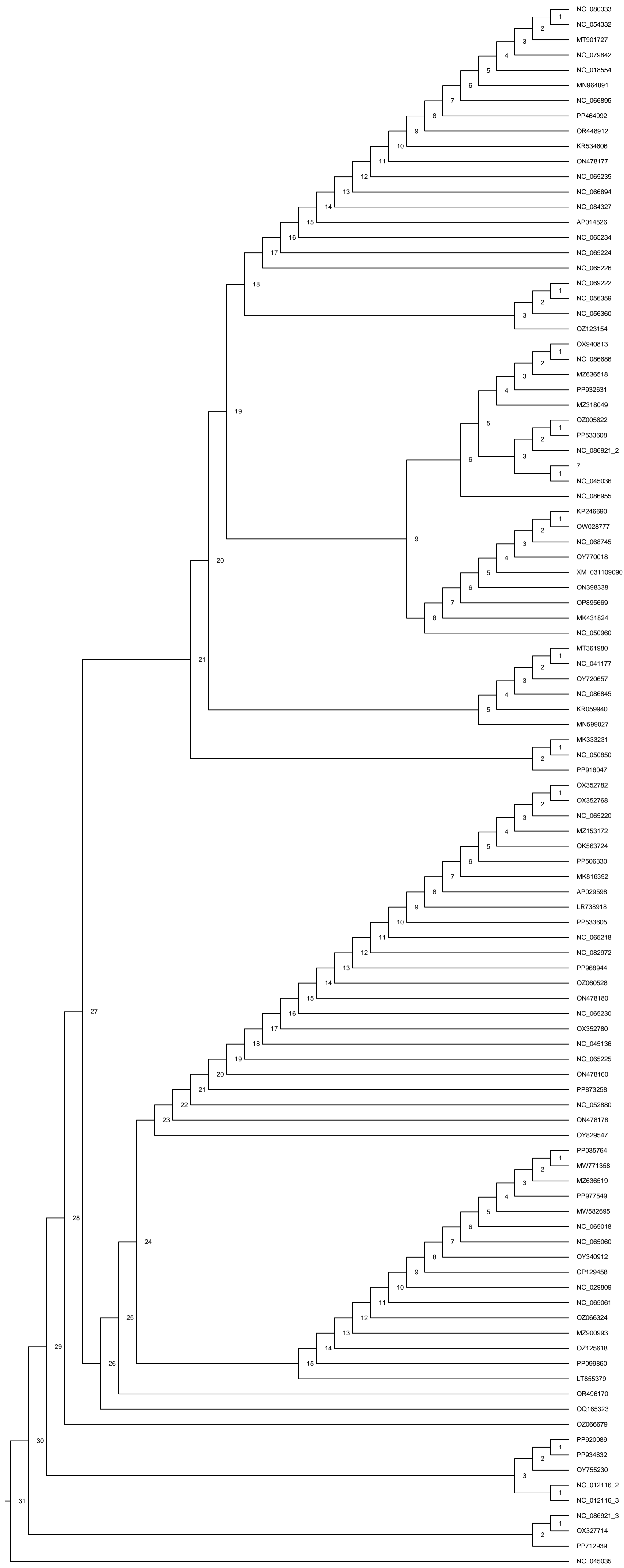

Supplement: Supplementary file 2 [file DataSheet2.zip › 补充图/Supplementary Fig 8.pdf]

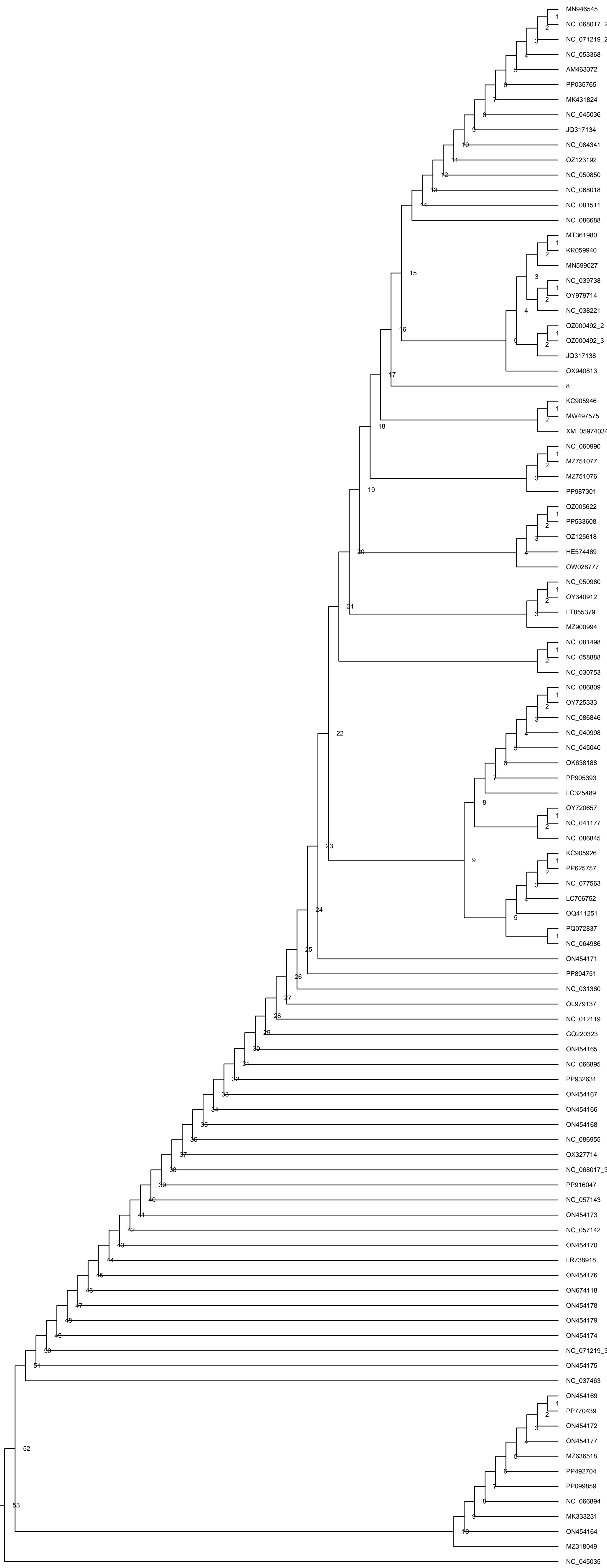

Supplement: Supplementary file 2 [file DataSheet2.zip › 补充图/Supplementary Fig 9.pdf]

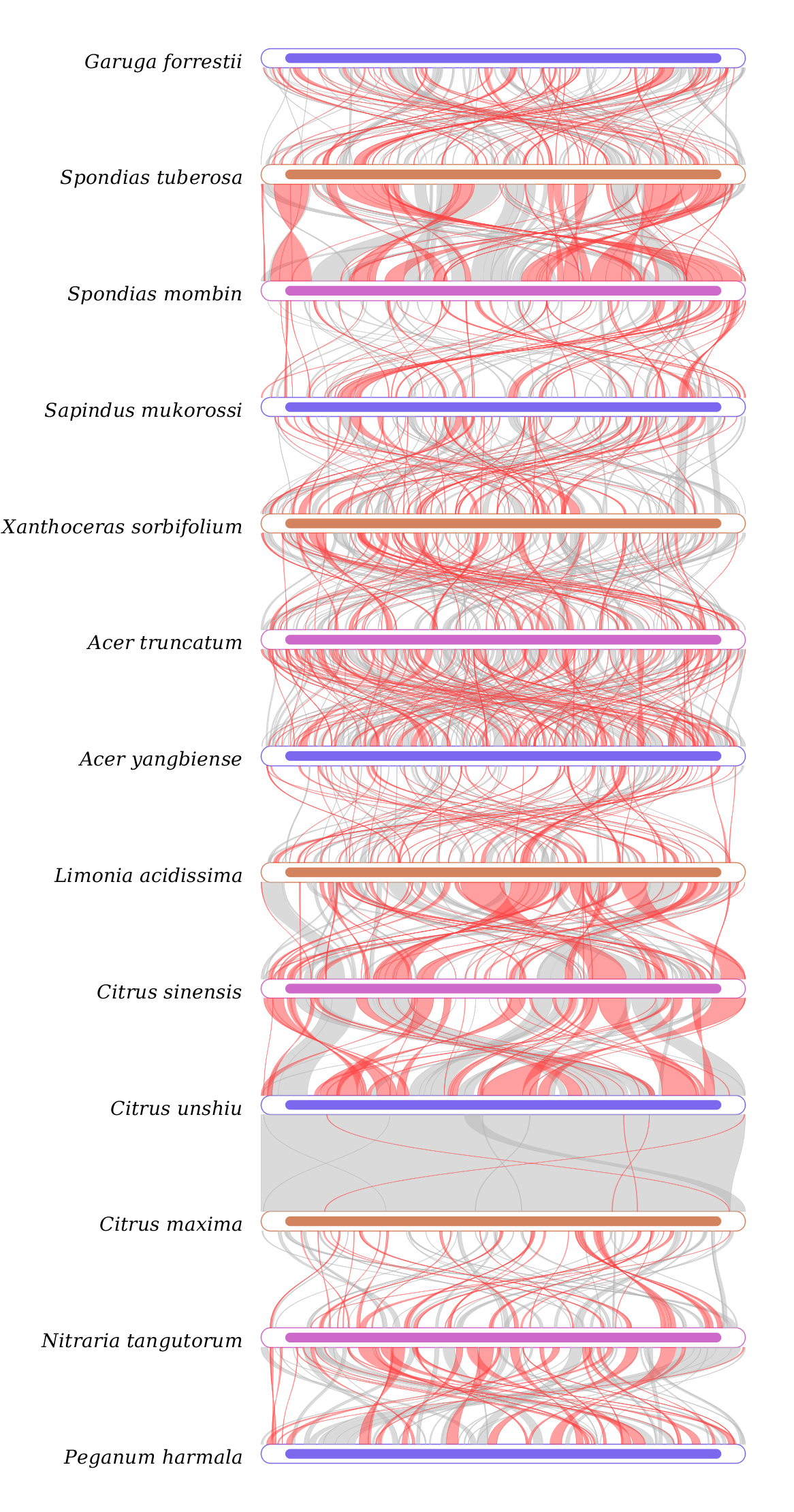

Supplement: Supplementary file 2 [file DataSheet2.zip › ▓╣│Σ═╝/Supplementary Fig 16.png]
